# Differential Effects of Psychotic Illness on Directed and Random Exploration

**DOI:** 10.1162/cpsy_a_00027

**Published:** 2020-08-01

**Authors:** James A. Waltz, Robert C. Wilson, Matthew A. Albrecht, Michael J. Frank, James M. Gold

**Affiliations:** 1 Maryland Psychiatric Research Center, University of Maryland School of Medicine, Baltimore, Maryland, USA; 2 Department of Psychology and Cognitive Science Program, University of Arizona, Tucson, Arizona, USA; 3 School of Public Health, Curtin Health Innovation Research Institute, Curtin University, Perth, Western Australia, Australia; 4 Department of Cognitive, Linguistic, and Psychological Sciences, Brown University, Providence, Rhode Island, USA; 5 Department of Psychiatry and Brown Institute for Brain Science, Brown University, Providence, Rhode Island, USA

**Keywords:** decision-making, ambiguity, reinforcement learning, schizophrenia, positive symptoms

## Abstract

Schizophrenia is associated with a number of deficits in decision-making, but the scope, nature, and cause of these deficits are not completely understood. Here we focus on a particular type of decision, known as the *explore/exploit* dilemma, in which people must choose between exploiting options that yield relatively known rewards and exploring more ambiguous options of uncertain reward probability or magnitude. Previous work has shown that healthy people use two distinct strategies to decide when to explore: directed exploration, which involves choosing options that would reduce uncertainty about the reward values (information seeking), and random exploration (exploring by chance), which describes behavioral variability that is not goal directed. We administered a recently developed gambling task designed to quantify both directed and random exploration to 108 patients with schizophrenia (PSZ) and 33 healthy volunteers (HVs). We found that PSZ patients show reduced directed exploration relative to HVs, but no difference in random exploration. Moreover, patients’ directed exploration behavior clusters into two qualitatively different behavioral phenotypes. In the first phenotype, which accounts for the majority of the patients (79%) and is consistent with previously reported behavior, directed exploration is only marginally (but significantly) reduced, suggesting that these patients can use directed exploration, but at a slightly lower level than community controls. In contrast, the second phenotype, comprising 21% of patients, exhibit a form of “extreme ambiguity aversion,” in which they almost never choose more informative options, even when they are clearly of higher value. Moreover, in PSZ, deficits in directed exploration were related to measures of intellectual function, whereas random exploration was related to positive symptoms. Taken together, our results suggest that schizophrenia has differential effects on directed and random exploration and that investigating the explore/exploit dilemma in psychosis patients may reveal subgroups of patients with qualitatively different patterns of exploration.

## INTRODUCTION

Growing evidence suggests that schizophrenia is associated with a number of decision-making deficits. In particular, relative to healthy volunteers (HVs), patients with schizophrenia (PSZ) show a reduced tendency to use potential reward magnitude in computing expected value (E. C. Brown et al., 2015; J. K. Brown et al., 2013), less systematic choice behavior (Strauss et al., 2011) and preferences (Strauss et al., 2012), and abnormal effort–cost computations in decision-making (Gold et al., 2013). However, these studies largely focus on the ability to maximize rewards, assuming the reward statistics are known (or learned from prior experience). Notably, choice of an option of lesser reward value is not always suboptimal, particularly if that choice would yield potentially useful information (i.e., by reducing the uncertainty about outcome statistics). Such *explore/exploit* choices arise frequently in daily life, whether it involves deciding between meals at a restaurant (explore the specials or exploit the pizza you know and love?) or treatments for a chronic disease (explore a novel treatment whose efficacy is uncertain or exploit a known treatment you have used for years?). From a computational perspective, however, striking the right balance between exploration and exploitation is difficult, as it involves trading off the relative benefits of information, which is useful for optimizing future decisions, and expected *value*, which is useful for making in-the-moment choices (Gittins, 1979; Gittins & Jones, 1979).

Recently, we have shown that HVs make explore/exploit decisions using a mixture of two behavioral strategies: *directed exploration*, in which information seeking drives exploration by choice, and *random exploration*, in which behavioral variability drives exploration by chance (Wilson, Geana, White, Ludvig, & Cohen, 2014). Algorithmically, directed and random exploration can be thought of as modifiers of the value associated with different options (which we denote as *Q*_*k*_ for the value of option *k*). In this framework, one can think of directed exploration as adding an *information bonus*, IB_*k*_, to the expected value of each option according to information provided by choosing that option (i.e., how much the outcome would reduce the uncertainty about the true expected value). In contrast, random exploration adds random *decision noise*, *n*_*k*_, to the values of both options. This noise tends to randomize the choice, making us more likely to explore low-value options by chance. Importantly, the level of randomness can be adjusted, as described below.

As an example, one way to decide between meals at a restaurant is to assign an expected value (*R*) to each option, corresponding to how rewarding we think each meal will be. Thus, a known quantity, pizza, might get a predicted value of *R*_pizza_ = 65 out of 100, indicating it has been reliably good in the past, whereas an unknown special (say, a new pasta dish) might get *R*_special_ = 50, based on our best guess about how good it will be. In this context, the known pizza receives no information bonus, IB_pizza_ = 0, because eating it tells us nothing new, whereas the pasta dish receives a large information bonus, IB_special_ = 20, because eating it is informative and we will learn how good it is. In contrast, random exploration simply adds noise to the value of all options, which will sometimes boost the value of exploring (e.g., *n*_pizza_ = 1, *n*_special_ = 15), but at other times may encourage us to exploit (e.g., *n*_pizza_ = 10, *n*_special_ = −5) or even to select an option that we think we know is bad (chopped liver for some; gefilte fish for others).

Previous work has indicated that directed exploration increases during adolescence, whereas random exploration remains constant (Somerville et al., 2017); that directed, but not random, exploration is reduced by inhibition of the frontal pole (Zajkowski, Kossut, & Wilson, 2017); and that random, but not directed, exploration is altered by the norepinephrine reuptake inhibitor atomoxetine (Warren et al., 2017). Importantly, a number of studies have found that schizophrenia is associated with increased aversion to various forms of uncertainty, including *risk*, where the probabilities of different outcomes are known (J. K. Brown et al., 2013), and *ambiguity*, where the probabilities of different outcomes are unknown (E. C. Brown et al., 2015; Cheng, Tang, Li, Lau, & Lee, 2012). Such uncertainty aversion works against directed exploration (where the more informative options by definition have greater potential for uncertainty reduction and thus are usually more uncertain to begin with).

While considerable evidence indicates that impaired reward-seeking behavior (goal-directed exploitation) may specifically contribute to negative symptoms (such as reduced motivation, or avolition) in schizophrenia (Strauss, Waltz, & Gold, 2014; Waltz & Gold, 2016), relatively few studies have examined whether motivational deficits in schizophrenia may be associated with deficits in directed *exploration*—the tendency to seek information in the service of resolving uncertainty. Consistent with this idea, in a response time task requiring participants to discover whether rewards were larger for faster or slower responses, PSZ—especially those with symptoms of anhedonia—showed reduced tendency to adjust their RTs toward the more uncertain outcomes (Strauss et al., 2011). This suggests that, even in the presence of intact reward sensitivity and reward-seeking behavior, avolition in schizophrenia could result from a reduced tendency to seek information in the service of resolving uncertainty. Although this result is consistent with reduced directed exploration, it could have resulted from increased uncertainty aversion writ large (i.e., regardless of exploration benefits) and also did not have such a straightforward approach to assessing random exploration. The latter point is particularly important given that a number of studies have found that schizophrenia is associated with increased baseline behavioral variability (Collins, Brown, Gold, Waltz, & Frank, 2014; Strauss et al., 2015), without assessing whether this variability is used for exploration.

Aside from exploration/exploitation, the issue of how PSZ balance prior beliefs and new evidence has recently become a major focus in the domain of perceptual processing. In that context, positive symptoms have been linked to an overweighting of prior beliefs relative to current sensory evidence, thereby distorting perceptual processing as well as belief formation (Baker, Konova, Daw, & Horga, 2019; Powers, Mathys, & Corlett, 2017). On the basis of evidence that uncertainty can drive exploration in the service of *reducing* uncertainty about an option, we speculated that if more severe psychosis is associated with greater reliance on prior beliefs, the severity of psychosis might also be linked to reduced flexibility in learning as well as a decrement in uncertainty-driven (directed) exploration. Moreover, if randomness is a strategy to compensate for the inability to direct exploration in the service of obtaining information and reducing uncertainty, it is possible that psychosis would also be linked to an *increased* tendency for random exploration.

**Figure 1.**
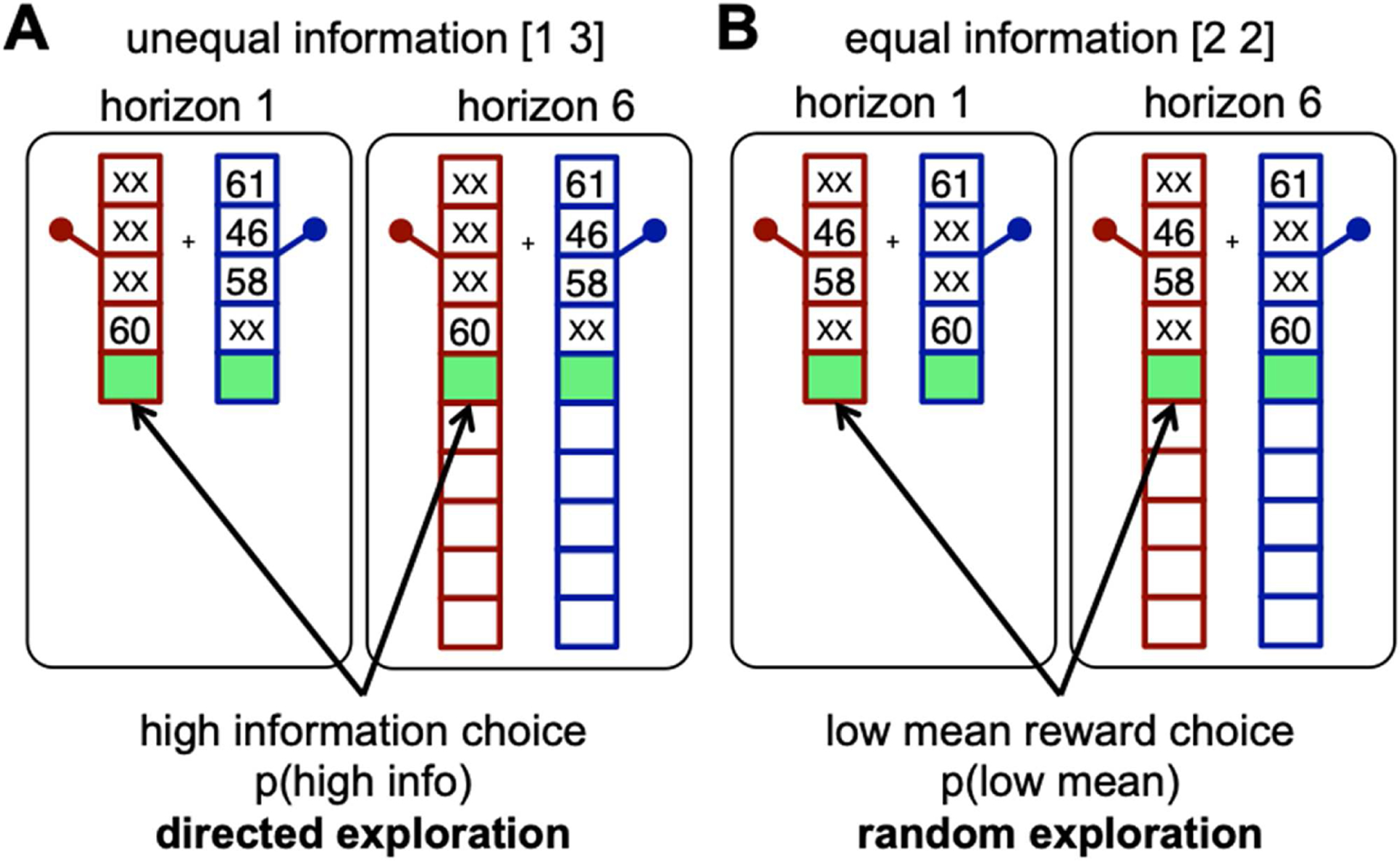
Schematic of the horizon and information conditions in the Horizon Task. In this task, participants play a series of 120 games, in a self-paced manner, lasting either 5 or 10 trials each, in which they choose between virtual slot machines, each of which pays out a reward in the form of points sampled from a Gaussian distribution whose mean is different for each option, varies from game to game, and is (initially) unknown to the subject. After four forced-choice trials, participants make either one or six free choices. The key manipulations in the Horizon Task are the number of free choices in each game (termed the “horizon”), which determines how valuable it is to explore, and the amount of information the participant has about each option (how many observed outcomes, from one to three). When the game is short (five total trials, one free choice; termed Horizon 1), exploration has no value since there is no opportunity to use new information in the future. When the game is long (10 total trials, 6 free choices; termed Horizon 6), it is often worth exploring at first to gain information that may be useful later on. The four forced-choice trials set up two information conditions: A) an unequal condition, or [1 3], in which subjects see one example from one bandit and three from the other, and B) an equal information, or [2 2], condition, in which subjects see two draws from each bandit. Thus, there are four combinations of horizons (1 vs. 6) and information (equal vs. unequal).

To determine whether schizophrenia was associated with changes in directed and random exploration, we used our recently developed behavioral task, known as the Horizon Task (Wilson et al., 2014), to quantify individual differences in directed and random exploration. In this task, participants make a series of decisions between virtual slot machines, loosely based on the “one-armed bandits” found in a casino, with participants receiving a bonus in proportion to the number of points they earn (Figure 1). When chosen, each bandit pays out a reward in the form of points sampled from a Gaussian distribution whose mean is different for each option, varies from game to game, and is (initially) unknown to the subject. Thus, to maximize their earnings, they must try to exploit the slot machine with the highest mean payoff but can only be sure which option is best by exploring first.

The key manipulation in the Horizon Task is the number of choices participants *will* make in the future—the time horizon. The horizon determines how valuable it is to explore. When the horizon is short, it is usually best to exploit (because any information value of the chosen option could not be further exploited in future trials); in contrast, when the horizon is long, exploration has more value. For example, when dining at a restaurant for the last time (a short horizon), one would likely exploit the favored pizza, but when expecting to return to a restaurant many times in the future (long horizon), one might take more time to explore the specials. In this way, the horizon manipulation allows us to quantify directed and random exploration as the *change* in information seeking and behavioral variability with horizon. Crucially, this manipulation allows us to distinguish directed and random exploration from baseline uncertainty attitude and baseline behavioral variability, factors that may be unrelated to exploration.

On the basis of our previous work (Strauss et al., 2011), we hypothesized that patients would show reduced directed exploration, relative to community controls. Moreover, we predicted that measures of directed exploration would correlate with the severity of negative symptoms like anhedonia and avolition (amotivation). By contrast, we predicted that measures of *random* exploration would *not* correlate with the severity of anhedonia and avolition, because these symptoms are thought to specifically reflect reductions in *goal-directed* behavior. That is, anhedonia and avolition are thought to specifically reflect reductions in goal-directed behavior and not necessarily increases in non-goal-directed behavior, and a reduction in goal-directed behavior does not necessarily *imply* an increase in non-goal-directed behavior (or vice versa). Finally, based on the idea that more severe psychosis is associated with greater reliance on prior beliefs, we predicted that some measures of exploration would correlate with the severity of *positive* symptoms.

## METHODS

### Participants

To determine the effect of psychotic illness on directed and random exploration, 108 people with a diagnosis of schizophrenia or schizoaffective disorder (referred to, collectively, as PSZ) and 33 healthy age-matched community volunteers (HVs) performed the Horizon Task at the Maryland Psychiatric Research Center (MPRC), University of Maryland School of Medicine. All participants gave informed consent, and the research was approved by the Institutional Review Boards at the University of Maryland School of Medicine.

### Clinical and Cognitive Measures

Patients were clinically and pharmacologically stable (no change in drug or dose for at least 4 weeks) outpatients from the MPRC or other nearby clinics. Almost all PSZ were being treated with antipsychotic medications (see Table S1 for details). The presence of a schizophrenia spectrum disorder in patients, as well as the absence of a current Axis I disorder (including drug dependence) and lifetime diagnosis of a psychotic disorder in HVs, was verified by screening with the Structured Clinical Interview for DSM–IV (First, Spitzer, Gibbon, & Williams, 1997). The absence of a neurological disorder, cognitively impairing medical disorder, and psychosis in first-degree relatives was verified by self-report. PSZ were further assessed with the Scale for the Assessment of Negative Symptoms (SANS; Andreasen, 1984) and the Brief Psychiatric Rating Scale (BPRS; Overall & Gorman, 1962).

PSZ and HVs were tested using a cognitive battery including the Wechsler Abbreviated Scale of Intelligence (WASI; Wechsler, 1999), the Wechsler Test of Adult Reading (WTAR; Wechsler, 2001), and the Measurement and Treatment Research to Improve Cognition in Schizophrenia (MATRICS) Consensus Cognitive Battery (MCCB; Green et al., 2004). There were significant differences between patients and community controls on all measures of cognition (Table 1).

### Experimental Task

We used the Horizon Task (Wilson et al., 2014; Figure 1) to quantify directed and random exploration. In this task, participants play a series of 120 games, in a self-paced manner, lasting either 5 or 10 trials each, in which they choose between virtual slot machines, each of which pays out a reward in the form of points sampled from a Gaussian distribution whose mean is different for each option, varies from game to game, and is (initially) unknown to the subject (the standard deviation, however, remained constant at 8 points). Specifically, in each game, the mean of one option (pseudorandomly chosen in a counterbalanced manner) was set to either 40 or 60 points, while the mean of the other was offset relative to this value by plus or minus 30, 20, 12, 8, or 4 points (pseudorandomly chosen in a counterbalanced manner). Participants were incentivized to earn as many points as possible, with points converted into money in a linear fashion (From the instructions: “The points you earn by playing the bandits will be converted into REAL money at the end of the experiment, so the more points you get, the more money you will earn.”) Participants were also instructed that one option was always better, in terms of expected value (“One of the bandits will always have a higher average reward”) and that the mean payout of each option was constant for each game.

To maximize their earnings, participants must exploit the slot machine with the higher average payoff, but they can only be sure which option is best by exploring first. The key manipulation in the Horizon Task is the number of trials in each game, the horizon, which determines how valuable it is to explore. When the horizon is short (one trial), exploration has no value, because there is no opportunity to use new information in the future. When the horizon is long (six trials), it is often worth exploring at first to gain information that may be useful later on. Thus, by contrasting behavior between Horizon 1 and Horizon 6 on the first choice of each game, the Horizon Task can quantify the components of behavior that are related to exploration.

To control the amount of information participants have before making a decision, each game starts with four forced-choice trials in which participants are instructed which option to choose. These forced trials set up two information conditions: an unequal condition, or [1 3], in which subjects are forced to play one bandit once (to obtain one example payout from that option) and the other bandit three times (to obtain three example payouts from that option) (Figure 1A), and an equal information, or [2 2], condition, in which subjects are forced to play both bandits twice (Figure 1B). Participants completed 30 games of each type (combination of horizon and information condition) for 120 games total, with the entire task taking roughly 50 minutes to complete. Basic performance on the task was quantified by computing the frequency with which participants chose the objectively correct option (i.e., the option with the higher generative mean), and participants were paid in proportion to the total number of points they earned.

### Measures of Directed and Random Exploration

The two information conditions in the Horizon Task allow us to quantify directed and random exploration in a model-free manner by looking at the first choice in each game, immediately after the four forced trials (Figure 1). Specifically, because directed exploration involves information seeking, it can be quantified as the probability of choosing the more informative option in the [1 3] condition, *p*(high info). Conversely, because random exploration involves decision noise, it correlates with the frequency of “errors,” choosing the low-mean option in the [2 2] condition, *p*(low mean). Crucially, computing these measures separately for each horizon condition allows us to (a) quantify baseline uncertainty seeking and behavioral variability as *p*(high info) and *p*(low mean) in Horizon 1 and (b) quantify directed and random exploration as the change in *p*(high info) and *p*(low mean) between Horizon 1 and Horizon 6.

### Statistical Analysis

To assess overall experimental task performance, we submitted individual accuracy scores (rates of choosing the option with the objectively higher mean) and response times to repeated-measures analyses of variance (ANOVAs), with choice (trial) number within a game as a within-subjects factor and diagnostic group as a between-subjects factor. We also performed repeated-measures ANOVAs to determine whether directed and/or random exploration varied as a function of horizon and diagnostic group. On the basis of our identification of a subgroup of participants showing extreme ambiguity aversion (AA; described below), we performed both of the above analyses on patient group with the addition of an AA group, as a factor. In cases of significant interactions, post hoc *t*-tests were used to assess differences in cell means. Additionally, we used *t*-tests and Mann–Whitney *U*-tests to examine effects of diagnostic group and AA group on measures intellectual function and symptom severity (depending on whether the scores were normally distributed). Finally, we used Spearman correlation analyses to assess relationships among experimental, standard cognitive, and clinical variables.

### Model-Based Analysis

**Figure 2.**
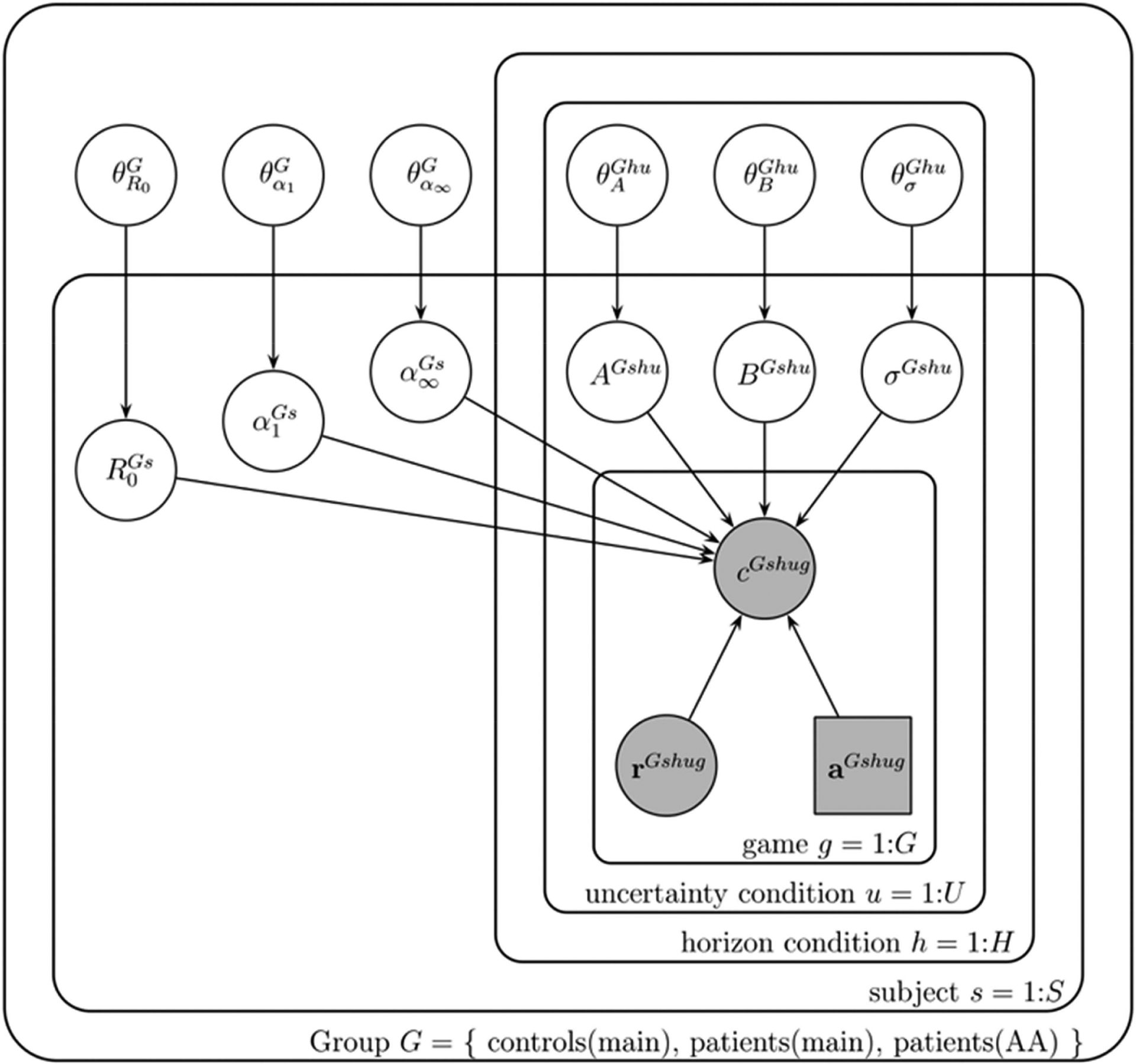
Graphical representation of the model. Each variable is represented by a node, with edges denoting the dependence between variables. Shaded nodes correspond to observed variables, that is, the free choices *c*^*Gshug*^, forced-trial rewards *r*^*Gshug*^, and forced-trial choices *a*^*Gshug*^. Unshaded nodes correspond to unobserved variables whose values are inferred by the model.

We modeled behavior on the first free choice of the Horizon Task using a slightly modified version of the Kalman filter model (Markov chain Monte Carlo method) presented in Zajkowski et al. (2017; Figure 2). This model assumes that participants use the outcomes of the forced-choice trials to learn an estimate of the mean reward of each option, as well as the uncertainty in their estimate of that mean, which is then fed into a decision rule that also includes terms for directed and random exploration.

Briefly, in this model, information seeking is quantified as an information weight, β_*I*_, with higher values of β_*I*_ corresponding to more information seeking. This allows us to quantify directed exploration as the change in information weight with horizon. Likewise, behavioral variability is quantified using a reward weight, β_*R*_, with higher reward weights associated with lower variability. This allows us to quantify random exploration as the change in reward weight with horizon.

In addition to quantifying directed and random exploration, the model allows us to quantify the learning process with three parameters: a prior mean, *R*_0_, and two learning rates corresponding to the initial learning rate, α_1_, and asymptotic learning rate, α_∞_. The initial learning rate parameter is related to the strength of the prior, with a lower value indicative of a stronger prior. Full details of the model and fitting procedure are outlined in the Supplementary Materials. In the next subsections, we describe the learning and decision-making components of the model in more detail.

### Learning Component

The learning component of the model assumes that participants learn the values for the mean reward of each option using the Kalman filter algorithm. The Kalman filter (Kalman, 1960) has been used to model learning in other learning tasks (Lee, Gold, & Kable, 2020) as well as other explore/exploit tasks (Daw, O’Doherty, Dayan, Seymour, & Dolan, 2006) and is a popular model of Bayesian learning, as it is both analytically tractable and easily relatable to the delta-rule update equations of reinforcement learning.

More specifically, the Kalman filter assumes a generative model in which the rewards from each bandit, *r*_*t*_, are generated from a Gaussian distribution with a fixed standard deviation, σ_*r*_, and a mean, $m_{t}^{i}$, that is different for each bandit, *i*, and can vary over time. The time dependence of the mean is determined by a Gaussian random walk with mean 0 and standard deviation σ_*d*_. Note that this generative model, assumed by the Kalman filter, is different from the true generative model used in the Horizon Task, in which the mean reward of each bandit is constant over time; that is, in the Horizon Task, σ_*d*_ = 0. This mismatch between the assumed and actual generative models is quite deliberate and allows us to account for the suboptimal learning of the subjects. In particular, this mismatch introduces the possibility of a recency bias (when σ_*d*_ > 0) whereby more recent rewards are overweighted in the model’s estimate of the mean reward, $R_{t}^{i}$, of each bandit. Note that $R_{t}^{i}$ corresponds to the estimated mean reward after *t* trials (i.e., after the model has seen *t* trials).

The actual equations of the Kalman filter model are straightforward. The model keeps track of an estimate of both the estimated mean reward, $R_{t}^{i}$, of each option, *i*, and the uncertainty in that estimate, $\sigma_{t}^{i}$. When option *i* is played on trial *t*, these two variables update according to

(1) $$R_{t}^{i}=R_{t}^{i}+{\left(\frac{\sigma_{t}^{i}}{\sigma_{r}}\right)}^{2}\left(r_{t}-R_{t-1}^{i}\right)$$

(2) $$\frac{1}{{\left(\sigma_{t}^{i}\right)}^{2}}=\frac{1}{{\left(\sigma_{t-1}^{i}\right)}^{2}+\sigma_{d}^{2}}+\frac{1}{\sigma_{r}^{2}}.$$

When option *i* is *not* played on trial *t*, we assume that the estimate of the mean stays the same but that the uncertainty in this estimate grows as the generative model assumes the mean drifts over time. Thus, for unchosen option *i*, we have

$$R_{t}^{i}=R_{t-1}^{i}\;\frac{1}{{\left(\sigma_{t}^{i}\right)}^{2}}=\frac{1}{{\left(\sigma_{t}^{i}\right)}^{2}+\sigma_{d}^{2}}.$$

When the option is played, the update equation (1) for $R_{t}^{i}$ is essentially just a “delta rule” (Rescorla & Wagner, 1972; Schultz,Dayan, & Montague, 1997), with the estimate of the mean being updated in proportion to the prediction error, $r_{t}-R_{t-1}^{i}$. This relationship to the reinforcement learning literature is made more explicit by rewriting the learning equations in terms of the time varying learning rate:

$$\alpha_{t}^{i}=\frac{{\left(\sigma_{t}^{i}\right)}^{2}}{\sigma_{T}^{2}}.$$

Written in terms of this learning rate, equations (1) and (2) become

$$R_{t}^{i}=R_{t-1}^{i}+\alpha_{t}^{i}\left(r_{t}-R_{t-1}^{i}\right)$$

and

$$\frac{1}{\alpha_{t}^{i}}=\frac{1}{\alpha_{t-1}^{i}+\alpha_{d}}+1,$$

where

$$\alpha_{d}=\frac{\sigma_{d}^{2}}{\sigma_{r}^{2}}.$$

The learning model has four free parameters: the noise variance, $\sigma_{r}^{2}$, the drift variance, $\sigma_{d}^{2}$, and the initial values of the estimated reward, *R*_0_, and uncertainty in that estimate, $\sigma_{0}^{2}$. In practice, only three of these parameters are identifiable from behavioral data, and we will find it useful to reparameterize the learning model in terms of *R*_0_ and an initial, α_1_, and asymptotic, α_∞_, learning rate. In particular, the initial value of the learning rate relates to σ_0_, σ_*r*_, and σ_*d*_ as

$$\alpha_{1}=\frac{\sigma_{0}^{2}+\sigma_{d}^{2}}{\sigma_{0}^{2}+\sigma_{d}^{2}+\sigma_{r}^{2}}.$$

While the asymptotic value of the learning rate, which corresponds to the steady state value of $\alpha_{t}^{i}$ if option *i* is played forever, relates to α_*d*_ (and hence σ_*r*_ and σ_*d*_) as

$$\alpha_{\infty }=\frac{1}{2}\left(-\alpha_{d}+\sqrt{\alpha_{d}^{2}+4\alpha_{d}}\right).$$

Although this choice to parameterize the learning equations in terms of α_1_ and α_∞_ is somewhat arbitrary, we feel that the learning rate parameterization has the advantage of being slightly more intuitive and leads to parameter values between 0 and 1, which are easier to interpret.

### Decision Component

Once the average reward of each option, $R_{t}^{i}$, has been estimated from the outcomes of the forced-choice trials, the model makes a decision using a simple logistic choice rule:

$$p(\text{choose right })=\frac{1}{1+\text{exp}\left(\beta_{R}\Delta R+\beta_{I}\Delta I+\beta_{S}\right)},$$

where $\Delta R=R_{t-1}^{\text{left }}-R_{t-1}^{\text{right }}$ is the *difference* in predicted reward between left and right options (note that the predicted reward for bandit *i* on time *t* is $R_{t-1}^{i}$) and Δ*I* is the difference in information between left and right options (which we define as +1 when left is more informative, −1 when right is more informative, and 0 when both options convey equal information in the [2 2] condition). Note that we code information, Δ*I*, as a categorical variable, not as a continuous variable. While in principle, Δ*I* should be a continuous variable, proportional to the uncertainty in each of the options $\sigma_{t}^{\text{left }}-\sigma_{t}^{\text{right }}$ (Gershman, 2019), in practice, the range of uncertainties in the Horizon Task is too small to dissociate the continuous from the categorical formulation.

The three free parameters of the decision process are the reward weight, β_*R*_, the information weight, β_*I*_, and the spatial bias weight, β_*S*_. Thus, the decision component of the model has 10 free parameters (β_*I*_ in the two horizon conditions, and β_*R*_ and β_*S*_ in the four Horizon × Uncertainty conditions). These parameters were fit for each subject using a hierarchical Bayesian approach outlined in detail in the Supplementary Material. Directed exploration is then quantified as the change in information weight with horizon, while random exploration is quantified as the change in reward weight with horizon. We assume that these three decision parameters can take on different values in the different horizon and uncertainty conditions (with the proviso that β_*I*_ is undefined in the [2 2] information condition, because Δ*I* = 0).

## RESULTS

### Overall Measures of Experimental Task Performance

#### Overall, HVs performed better than PSZ, but this difference in performance did not change over the course of the game

**Figure 3.**
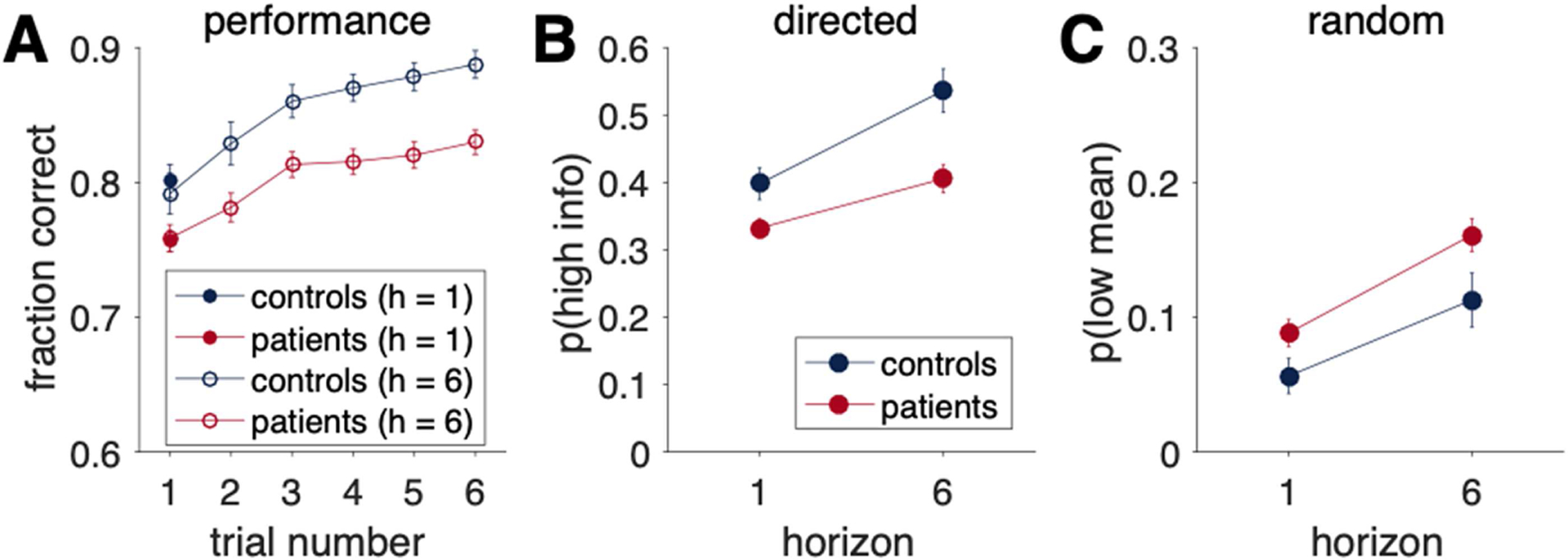
Performance on the Horizon Task by diagnostic group. A) Proportions of optimal responses as a function of trial number for Horizon 1 (filled circles) and Horizon 6 (open circles) games. B, C) Model-free analysis of the first free choice as a function of horizon, with B showing proportions of high-information (information seeking) choices and C showing proportions of low-mean choices (indicative of behavioral variability).

All groups chose the objectively correct (higher value) option more often than would be predicted by chance (controls, mean fraction correct = 0.71; patients, 0.68), both *p*s < 0.001. Participants also showed evidence of learning, manifest by an increase in performance as a function of time (Figure 3A), *p* < 0.001, that was qualitatively consistent with the previously reported behavior of healthy young adults (Wilson et al., 2014). Overall, HVs performed better than PSZ, as indicated by a main effect of group, *F*_1,695_ = 8.53, *p* = 0.004, but this difference in performance did not change over the course of the game (interaction between group and trial number, *F*_5,695_ = 1.11, *p* = 0.35).

### Measures of Directed and Random Exploration

#### PSZ showed reduced directed exploration relative to HVs

As noted above, our main analyses (repeated-measures ANOVAs with horizon as a within-subjects factor and group as a between-subjects factor) focused on behavior on the first free choice as a function of horizon. These analyses revealed that patients with schizophrenia showed reduced directed exploration, relative to HVs, as main effects of group, *F*_1,139_ = 8.83, *p* = 0.003, and horizon, *F*_1,139_ = 68.2, *p* < 0.001, on *p*(high info) were qualified by a significant Group × Horizon interaction, *F*_1,139_ = 6.41, *p* = 0.012. As shown in Figure 3B, PSZ showed lower information seeking during Horizon 6 games, *t*_139_ = 3.20, *p* = 0.002, and, to a lesser extent, during Horizon 1 games, *t*_139_ = 2.26, *p* = 0.026. Moreover, PSZ showed a reduction in the change in *p*(high info) with horizon, *t*_139_ = 2.53, *p* = 0.012, consistent with less directed exploration in patients.

#### PSZ and HVs did not differ in random exploration

Consistent with prior reports, the entire sample of participants showed increased behavioral variability with horizon (increases in low-mean value choices with increased uncertainty), *F*_1,139_ for main effect of horizon = 44.91, *p* < 0.001. Furthermore, patients showed greater overall behavioral variability relative to controls, *F*_1,139_ for main effect of group = 3.99, *p* = 0.05. However, as shown in Figure 3C, the two groups did not differ in the impact of horizon on behavioral variability, as the interaction between horizon and group was not significant, *F*_1,139_ = 0.70, *p* = 0.403. Thus, there was no evidence that the two groups differed in their levels of random *exploration*.

### Identification of Patient Subgroups Based on Ambiguity Aversion

#### Twenty-one percent of patients exhibit a form of extreme ambiguity aversion, in which they almost never chose more informative options

**Figure 4.**
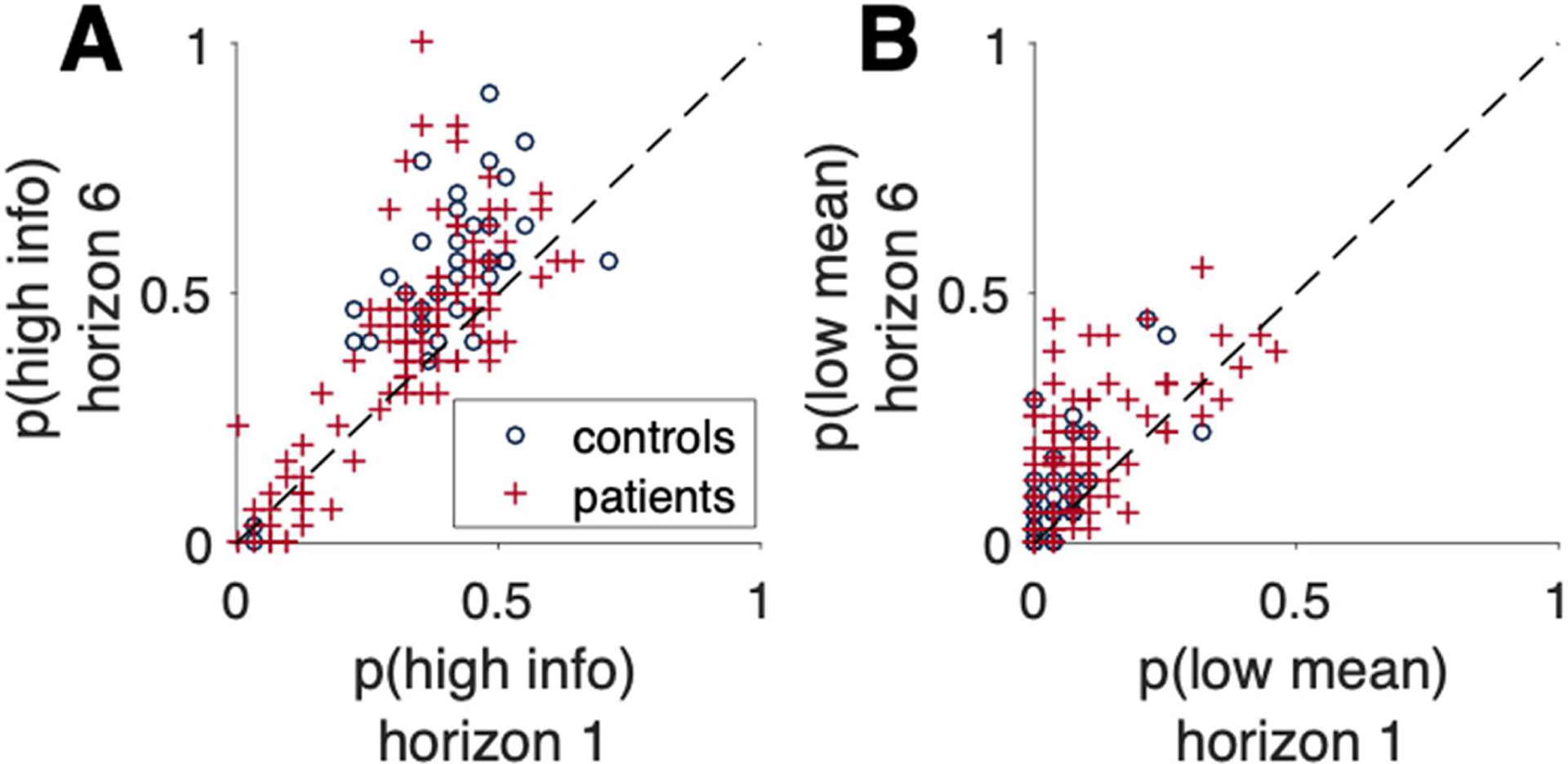
Percentages of information choices. A) Percentages of high-information choices at Horizon 1 plotted against those at Horizon 6, in patients (red crosses) and controls (blue circles). While most participants make more high-information choices at Horizon 6 than at Horizon 1, a subset of participants (predominantly schizophrenia patients) make few high-information choices in both horizon conditions. These individuals were said to be “ambiguity averse.” B) Percentages of low-mean-value choices at Horizon 1 plotted against those at Horizon 6, in patients (red crosses) and controls (blue circles).

In addition to examining between-group differences in directed and random exploration, we looked at individual differences in directed and random exploration. In Figure 4A, we plot *p*(high info) in Horizon 6 against *p*(high info) in Horizon 1. In Figure 4B, we do the same thing for *p*(low mean). In these plots, each point corresponds to a participant, and the diagonal line is the line of equality. Thus, points above the diagonal line correspond to subjects showing increased directed (90.9% community controls, 73.1% patients) or random (66.7% community controls, 67.6% patients) exploration with horizon. The most striking feature of Figure 4A is the separation of two groups of subjects with regard to directed exploration: a group of 25 participants showing extreme ambiguity aversion [*p*(high info) < 0.25 for both Horizon 1 and Horizon 6], at the bottom left, and a group of non-AA participants, in the center of the plot, accounting for the majority of participants from both diagnostic groups (93.9% community controls, 78.7% patients), exhibiting behavior similar to what we have previously seen in students and younger teens (Somerville et al., 2017; Wilson et al., 2014). This clustering into two groups based on *p*(high info) was supported by additional clustering analysis using *k*-means and Gaussian mixture model analysis (see Supplementary Materials for details). In brief, our by-eye heuristic was more conservative than k-means (which puts two more subjects in the AA group) and less conservative than Gaussian mixtures (which puts three fewer subjects in the AA group). For this reason, we retained the original heuristic as the cutoff in the article. Regardless of the clustering, the qualitative results are the same.

Participants in the AA group almost never chose the more ambiguous (and hence higher information) option. This group of 25 individuals has many more patients (*n* = 23, 21.3% of patients) than community controls (*n* = 2, 6.1% of community controls). Such extreme ambiguity aversion has not been seen before in either student or adolescent populations in this same task (Somerville et al., 2017; Wilson et al., 2014).

**Figure 5.**
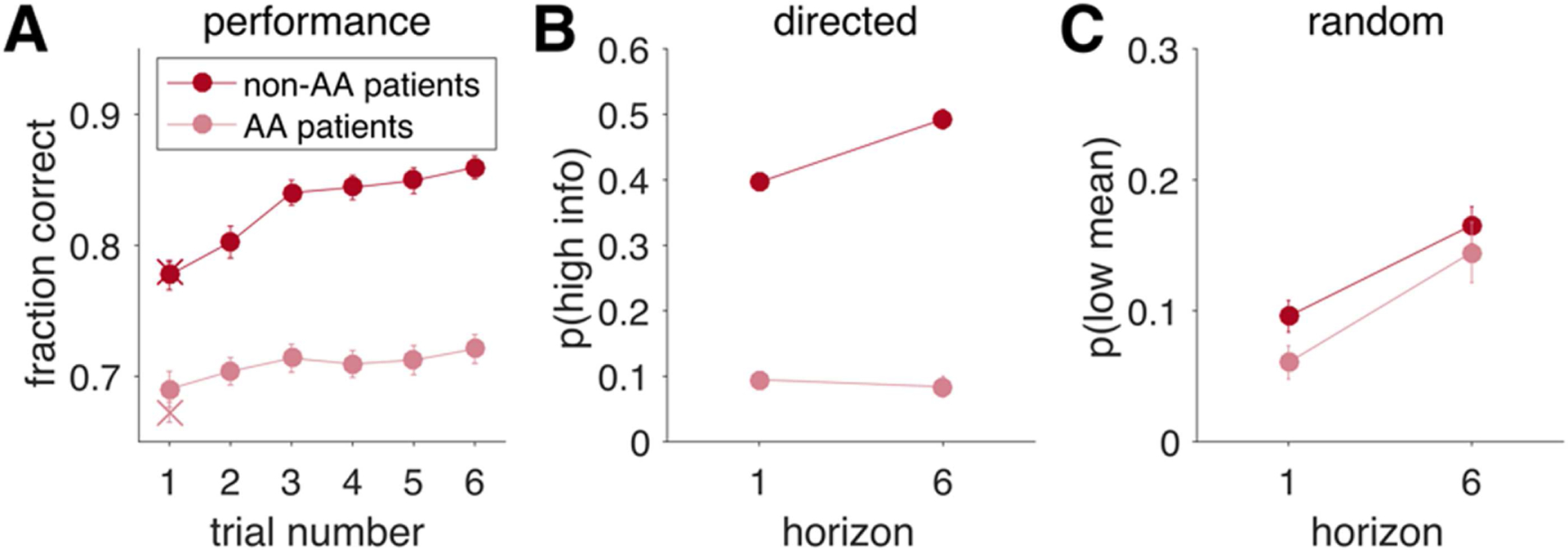
Comparison of experimental measures from the Horizon Task in ambiguity-averse (AA) and non-AA patients. A) Overall Horizon Task performance in AA and non-AA patients. B) Directed Exploration in AA and non-AA patients. C) Random Exploration in AA and non-AA patients.

AA had a clear impact on performance, as AA patients chose the objectively high reward option much less frequently than non-AA patients, *t*_106_ = 6.86, *p* < 0.001 (Figure 5A). In addition, AA patients exhibited impaired learning over the course of the game, *F*_5,530_ for Group × Trial Number = 4.32, *p* < 0.001. Nevertheless, overall performance in AA patients was above chance, indicating at least some engagement with the task (mean fraction correct = 0.63), t-test relative to chance *t*_22_ = 21.10, *p* < 0.001. Critically, however, despite large differences in measures of directed exploration (by definition, and see Figure 5B), AA and non-AA patients did not differ on measures of random exploration, *t*_106_ = 1.140 for Σ*p*(low mean), *t*_106_ = −0.614 for Δ*p*(low mean; Figure 5C). Both groups showed evidence of an effect of horizon on random exploration, with increased variability for Horizon 6 compared to Horizon 1, non-AA *t*_84_ = 6.18, *p* < 0.001; AA *t*_22_ = 4.53, *p* < 0.001.

Model-based analyses further reinforced the above interpretation, revealing that AA patients showed no evidence for information seeking in any context. Consistent with model-free measures of directed and random exploration, model-based analyses indicated that AA patients showed no evidence for information seeking in any context (Figure S3). Specifically, for the AA patients, we observed reduced information weighting in both Horizon 1 and Horizon 6 relative to non-AA group patients (100% of samples less than zero, respectively). In addition, AA patients showed much reduced reward weight in the [1 3] condition (100% of samples), which is almost zero for most people in this group. Both results are consistent with the extreme AA in this group and suggest that AA patients base their decisions almost exclusively on avoiding the uncertain option in the [1 3] condition. Interestingly, reward weight does not appear to differ between non-AA and AA patients in the [2 2] condition, consistent with the ability of AA patients to perform quite well in this condition.

#### Among non-AA participants, there was a trend toward a main effect of diagnostic group on performance

Although the main group of (*n* = 85) non-AA patients showed evidence for directed exploration (effect of horizon on choice of informative option *t*_84_ from one-sample test on Δ*p*(high info) = 6.71), *p* < 0.001, their levels of overall performance, *t*_114_ = −2.49, *p* = 0.015, and directed exploration, *t*_114_ for between-group difference in Δ*p*(high info) = −2.454, *p* = 0.013, there was a trend toward a main effect of diagnostic group on performance, *F*_1,570_ = 3.67, *p* = 0.058 (Figure S4A), suggestive of reduced performance relative to community controls. Among non-AA participants, the interaction between group and horizon was not significant, *F*_5,570_ = 0.57, *p* = 0.721, however.

Next, we asked whether non-AA controls and non-AA patients showed differential effects of horizon on directed exploration, finding that the interaction between group and horizon trended toward significance, *F*_1,114_ = 2.81, *p* = 0.059 (Figure S4B). A post hoc *t*-test directly comparing non-AA patients and non-AA controls revealed a significant between-group difference in the proportion of high-information choices at Horizon 6, two-sided *t*_114_ = 2.69, *p* = 0.008. There was also a trend toward significantly elevated levels of behavioral variability in non-AA patients relative to non-AA controls, *F*_1,114_ for main effect of diagnosis on Σ*p*(low mean) = 3.75, *p* = 0.055 (Figure S4C). However, the lack of a significant interaction between subject type and horizon, *F*_1,114_ = 0.20, *p* = 0.66, indicates that non-AA patients, relative to non-AA controls, did not differ in random exploration.

Consistent with the model-free measures, non-AA patients exhibited reduced directed exploration relative to controls, as indicated by a reduction in the information weight, β_*I*_, in Horizon 6 (96.5% of samples for the mean information weight of non-AA group patients below those for non-AA group controls; Figure S5). As expected, non-AA patients and non-AA controls differed in their levels of educational attainment and on multiple measures of cognitive performance (Table S1).

#### In PSZ, information-seeking behavior was related to measures of intellectual function

Other than task performance, what distinguished AA patients from non-AA patients? Surprisingly, we found that the two patient subgroups identified based on AA scores did not differ on any symptom measure (effect sizes *d* = −0.23–0.17), including ratings for avolition/anhedonia, *t*_106_ = 0.557. The two groups differed greatly, however, on measures of intellectual function, including estimates of current IQ (from the WASI) and premorbid IQ (from the WTAR and the Reading subtest of the WRAT; see Table 2). Furthermore, we observed significant differences between AA and non-AA patients on composite scores and working memory domain scores from the MATRICS battery, *t*_106_ = 2.53, *p* = 0.013.

### Relationships Among Measures of Explore/Exploit Behavior, Cognition, and Symptoms

#### In PSZ, random exploration was related to positive symptom severity

**Figure 6.**
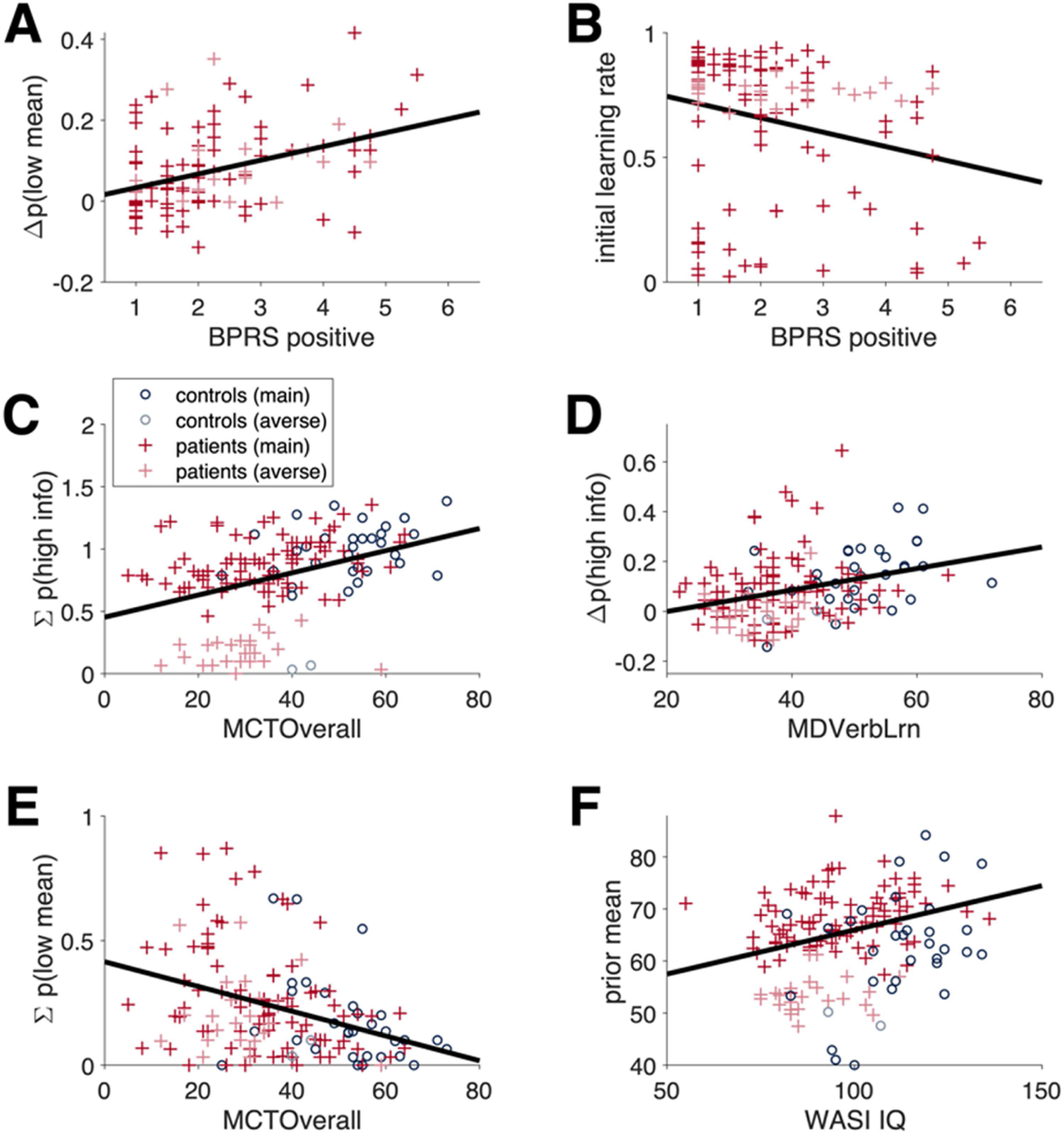
Relationships between cognitive and behavioral measures in patients. A) Composite Measurement and Treatment Research to Improve Cognition in Schizophrenia (MATRICS) scores correlated positively with overall information seeking in both patients and controls. B) Composite MATRICS scores correlated inversely with overall behavioral variability in both patients and controls. C) Verbal Learning scores from the MATRICS correlated positively with directed exploration scores in both patients and controls. D) Overall IQ estimates from the Wechsler Abbreviated Scale of Intelligence correlated positively with prior mean, a model-based performance metric. Also in patients, ratings for psychotic symptoms correlated (E) positively with random exploration scores and (F) negatively with initial learning rate, a model-based indicator of the strength of prior beliefs.

Finally, we looked at the correlations between cognitive and symptom measures and both the model-free and model-based measures of behavior. We observed one systematic relationship between a clinical symptom measure and a measure of information seeking: Mean negative symptom scores correlated negatively with change in information weight, a model-based measure of directed exploration, such that patients with higher levels of negative symptoms showed reduced directed exploration (Table 3). We observed several significant correlations between clinical symptom severity and measures of behavioral variability (random exploration). Specifically, we observed a significant correlation between the model-free measure of behavioral variability [Σ*p*(low mean)] and the positive symptom cluster score from the BPRS, rho_106_ = 0.23, *p* = 0.015. We also observed significant correlations between the model-free measure of random exploration [Δ*p*(low mean)] and both the positive symptom cluster score from the BPRS, rho_106_ = 0.39, *p* < 0.001 (Figure 6A) and the overall BPRS score, rho_106_ = 0.29, *p* = 0.002. We observed significant negative correlations between positive symptom scores from the BPRS and several model-based measures of behavioral variability and random exploration (Table 3). Finally, we observed a significant negative correlation between a model-based measure of performance (the initial learning rate, α_1_) and both the positive symptom cluster score from the BPRS and the overall BPRS score (Figure 6B). As noted in the Methods, the initial learning rate parameter is related to the strength of the prior, with a lower value indicative of a stronger prior. Thus, a significant negative association between the learning rate and positive symptoms is especially intriguing, as it implies that increased positive symptoms are associated with a stronger effect of the prior (indicated by lower α_1_). This finding is consistent with the results of several earlier studies (Baker et al., 2019; Powers et al., 2017) as well as theoretical accounts of inference in schizophrenia (Corlett et al., 2019; Fletcher & Frith, 2009; Jardri & Deneve, 2013), suggesting that positive symptoms are related to (and perhaps even caused by) overly strong priors. The presence of overly strong priors in a subset of patients may explain why patients showed similar rates of random exploration, but reduced rates of directed exploration, relative to controls.

#### Correlations between cognitive variables and behavior in the patient group

As shown in Table 4, we observed multiple significant correlations between standardized measures of intellectual function and experimental measures of information seeking. Overall information seeking [Σ*p*(high info)] across the two-horizon condition correlated strongly with numerous measures of intellectual function, including estimates of current and premorbid IQ (from the WASI and WTAR, respectively), a measure of cross-domain cognitive capacity (the MATRICS composite scores; Figure 6C), and measures of domain-specific abilities, from the MATRICS (Working Memory, Processing Speed, Attention and Vigilance, and Verbal Learning). With all of these measures, patients with evidence of higher intellectual capacity showed greater overall information-seeking behavior. Horizon-dependent increases in directed exploration [as measured by Δ*p*(high info)] also correlated with several measures of intellectual function: estimated current IQ from the WASI, MATRICS composite score, and MATRICS Verbal Learning (Figure 6D); that is, patients with higher intellectual capacity were more likely to exhibit adaptive changes in information-seeking behavior, with longer horizon.

It is important to note that almost all measures of cognitive function showing positive correlations with information-seeking behavior showed negative correlations with undirected behavioral variability, as measured by Σ*p*(low mean; see Table 4 and Figure 6E). Intellectual variables in controls were *also* found to correlate positively with information-seeking behavior and negatively with behavioral variability (see the Supplementary Results and Table S3). Finally, multiple strong correlations were also observed between standard cognitive measures and model-*based* measures of performance in patients (Table S4). Specifically, patients exhibited a significant correlation between a model-based measure of overall performance (the prior mean parameter) and overall estimated IQ, from the WASI (Figure 6F). This same relationship was observed in controls (Table S5).

## DISCUSSION

In this article, we investigated the explore/exploit trade-off in patients with schizophrenia. In particular, we asked whether patients differed from community controls in their tendency to engage in directed and random exploration and in their overall level of uncertainty aversion and behavioral variability. We found that patients, as a group, showed reduced information seeking, reduced horizon-dependent directed exploration, and increased overall behavioral variability, but no difference in horizon-dependent random exploration. That patients were sensitive to horizon for random exploration is indicative that they understood the task and acted appropriately when exploration was of more potential utility; they simply did not show the same degree of directed/strategic exploration for information gain.

Indeed, a major driver of the difference between PSZ and HVs was the prevalence of extreme ambiguity aversion in the PSZ population. Participants in this ambiguity-averse group almost never chose the more informative option on the first, or later, free choice trials. Such behavior was rare in the HVs recruited for this study (only 2/33 showed extreme AA) and was not apparent in previously reported studies in college students and adolescents (Somerville et al., 2017; Wilson et al., 2014). Separating our subjects into three subgroups (non-AA HVs, non-AA PSZ, and AA PSZ—the AA HV group was too small for meaningful analysis), we found that directed exploration was absent in the AA PSZ, yet still significantly diminished in non-AA PSZ, relative to non-AA HVs. In contrast, there were no differences in random exploration across the three groups. This suggests that schizophrenia has a selective impact on directed exploration. Directed exploration has been shown to rely predominantly on prefrontal cortical mechanisms (Daw et al., 2006), with rostrolateral PFC having been identified as one specific locus (Badre, Doll, Long, & Frank, 2012; Zajkowski et al., 2017). Importantly, prefrontal processes underlying uncertainty processing and decision-making have frequently been implicated in abnormalities in learning and decision-making in schizophrenia (Hernaus et al., 2018; Krug et al., 2014; Lancaster et al., 2016). If extreme ambiguity aversion does reflect a qualitatively different decision-making process, then a key goal for future work should be to determine exactly what that difference entails, from both cognitive and neural perspectives. Exploratory analyses of the current data set found no differences in symptom measures in the AA PSZ group and the main group of PSZ, but these groups did differ on measures of current and premorbid IQ, as well as current working memory function.

Furthermore, we observed a number of significant correlations between experimental measures of behavior and symptom scores. Of particular interest was the negative correlation between positive symptoms and the initial learning rate in the model. A higher initial learning rate in PSZ with greater positive symptoms is consistent with the idea that more psychotic patients use stronger prior beliefs (Baker et al., 2019; Powers et al., 2017). According to our framework, the strength of priors is negatively correlated with learning rate, such that overly strong priors would be associated with a reduced impact of PEs and reduced updating. Thus, it would take many more large PEs to adapt the posterior. This is because the strength of the prior is represented by the variance about the mean expected value, rather than the mean itself and is thus more likely to influence the impact of the prediction error (the learning rate) than the magnitude of the prediction error.

We also found that measures of random, but not directed, exploration showed positive correlations with psychotic symptom severity, such that more psychotic patients exhibited more random exploration. This result suggests that more psychotic patients may exhibit more unsystematic behavioral variability, in conditions with greater uncertainty (i.e., longer horizons), as a compensatory mechanism for an overall reduced ability/tendency to engage in greater information-seeking behavior. Further tests of these ideas could come from studies using measures of psychosis-like phenomena in healthy individuals, such as the Peters Delusions Inventory (Peters, Joseph, Day, & Garety, 2004) or the Cardiff Anomalous Perceptions Scale (Bell, Halligan, & Ellis, 2006), or in studies involving samples of psychosis patients and controls, where symptom data are available for both groups.

Contrary to our hypotheses, we did *not* observe strong correlations between experimental measures of exploration and negative symptom severity. In our previous work (Strauss et al., 2011), we observed a significant correlation between the severity of clinically rated anhedonia and a computational measure of uncertainty-driven exploration. However, that task was not designed to dissociate uncertainty *aversion* from reduced directed exploration, per se. The results of the current study suggest that uncertainty aversion may contribute substantially to deficits in goal-directed exploration, in psychotic illness. Of note, we also did not observe strong correlations between negative symptom severity and experimental measures of *random* exploration, in either a positive or negative direction; that is, the effect of uncertainty (horizon) on the tendency to engage in unsystematic behavioral variability was not a function of the severity of motivational deficits in PSZ. Whereas measures of directed and random exploration were found to be largely unrelated to measures of motivational deficits in PSZ, we observed numerous systematic relationships between measures of exploration and measures of intellectual function. Specifically, we found that most cognitive measures correlated positively with measures of directed exploration but negatively with measures of random exploration. These results indicate that strategic information-seeking behavior is most characteristic of individuals with the greatest capacity to *use* information to guide learning and behavior. By contrast, random exploration was most prominent in PSZ with the most severe cognitive impairment. In short, a critical manner in which schizophrenia impacts learning is by reducing information-seeking behavior. The question of whether reduced information-seeking behavior is a cause or consequence of deficits in working memory, selective attention, and processing speed, for example, has not been resolved, however.

### Study Limitations

Our study had a number of limitations that could affect the generalizability of our results. Specifically, the gambles in the Horizon Task involved only gains and no losses. Thus, we cannot discern whether such a large group of participants would show extreme ambiguity aversion with regard to potential losses.

Second, one might question whether the extreme ambiguity-aversion behavior reflects a qualitatively different decision-making process, such as a failure to understand the task. While the present data cannot rule out this interpretation, we believe that it is unlikely to account for the observed effects. Of note, participants in the AA group performed particularly well, if not *better* than controls, in the *equal* information condition (i.e., when there was no difference in uncertainty between the options). Thus, participants in the AA group were able to choose the more rewarding option very effectively when the estimation of expected value depended solely on the integration of previous outcomes and was not influenced by the *amount* of information about either option. Nonetheless, task performance in PSZ may have been aided by additional training or informational displays designed to reduced working memory demands, and future studies on this topic should include the use of debriefing methods, such as quizzes, to identify participants who fail to fully comprehend the task and exhibit poor performance for nonspecific reasons.

Third, while correlation analyses revealed no significant effects of antipsychotic medication type or dosage on any of our dependent variables, definitively identifying or ruling out influences of psychotropic medications on experimental measures of exploration (especially given known effects of norepinephrine-modulating drugs on exploratory behavior; Warren et al., 2017) would require either studies involving antipsychotic-naive patients or controlled clinical trials.

### Summary

In summary, our findings suggest that schizophrenia has dissociable effects on directed exploration and random exploration. Such a dissociation between the two types of exploration is consistent with a number of our earlier findings (Somerville et al., 2017; Warren et al., 2017; Zajkowski et al., 2017). While the full extent of the neural circuits underlying directed and random exploration is currently unknown, a key question for future work will be to determine whether the changes in explore/exploit behavior seen here in PSZ are related to specific aspects of brain function.

## Supplementary Material

Supplementary information

## Figures and Tables

**Table 1.** Demographic, cognitive, and clinical measures in the full sample of patients and controls

Measure	Patients^a^	Controls^b^	Inferential statistic
Demographic			
Age, years, *M(SD)*	37.0 (10.1)	36.4 (10.4)	*t* = 0.307
Gender	36 female, 72 male	11 female, 22 male	*χ*^2^ = 0.000
Race	53 C, 43 AFA, 4 AS, 7 M/O	18 C, 13 AFA, 0 AS, 2 M/O	*χ*^2^ = 1.372
Subject education, *M*(*SD*)	13.2 (2.1)	15.1 (2.1)	*t* = 4.374***
Parental education, *M*(*SD*)	14.3 (2.9)	14.0 (2.6)	*t* = 0.671
Cognitive, *M(SD)*			
WASI estimated IQ (four subtests)	94.5 (14.1)	111.5 (13.8)	*t* = 6.05***
WRAT-Reading scaled score	97.2 (14.8)	109.7 (15.0)	*t* = 4.26***
WTAR scaled score	99.0 (17.5)	110.9 (14.0)	*t* = 4.01***
MATRICS composite score	32.9 (12.7)	51.3 (11.0)	*t* = 7.56***
MATRICS domain scores			
Processing Speed	38.1 (12.5)	53.1 (11.8)	*t* = 6.10***
Attention/Vigilance	39.9 (11.3)	52.3 (11.2)	*t* = 5.53***
Working Memory	39.7 (10.4)	51.9 (11.6)	*t* = 5.74***
Verbal Learning	37.7 (7.9)	50.6 (8.8)	*t* = 8.01***
Visuospatial Learning	36.0 (12.3)	45.5 (10.7)	*t* = 3.99***
Reasoning/Problem Solving	43.4 (10.8)	49.6 (9.8)	*t* = 2.98**
Social Cognition	41.9 (12.0)	54.6 (8.0)	*t* = 5.70***
Clinical, *M*(*SD*)			
BPRS mean item score—all items	1.7 (0.4)		
BPRS mean item score—Psychosis	2.2 (1.2)		
BPRS mean item score—Depression	1.9 (0.9)		
SANS mean item score—all items	1.5 (0.7)		
SANS mean item score—Avolition/Anhedonia	2.0 (0.9)		

*Note.* AFA = African American. AS = Asian. BPRS = Brief Psychiatric Rating Scale. C = Caucasian. MATRICS = Measurement and Treatment Research to Improve Cognition in Schizophrenia Consensus Cognitive Battery. M/O = mixed/other. SANS = Scale for the Assessment of Negative Symptoms. WASI = Wechsler Abbreviated Scale of Intelligence. WRAT-Reading = Wide-Ranging Achievement Test, Reading Subtest. WTAR = Wechsler Test of Adult Reading.

a *n* = 108.

b *n* = 33.

* *p* < 0.05.

** *p* < 0.01.

*** *p* < 0.001.

**Table 2.** Demographic, cognitive, and clinical measures in patient subgroups

Measure	Non-AA patients^a^	AA patients^b^	Inferential statistic
Demographic			
Age, years, *M*(*SD*)	36.7 (10.3)	38.3 (9.6)	*t* = 0.672
Gender	26 female, 59 male	10 female, 13 male	*χ*^2^ = 1.353
Race	47 C, 27 AFA, 3 AS, 7 M/O	6 C, 16 AFA, 1 AS, 0 M/O	*χ*^2^ = 11.489**
Subject education, M (SD)	13.3 (2.1)	13.0 (2.1)	*t* = 0.675
Parental education, M (SD)	14.6 (2.7)	13.5 (3.4)	*t* = 1.611
Cognitive, *M*(*SD*)			
WASI estimated IQ (four subtests)	96.1 (14.7)	88.9 (10.0)	*t* = 2.721**
WRAT-Reading scaled score	98.6 (15.5)	91.8 (10.5)	*t* = 2.457*
WTAR scaled core	101.0 (17.6)	91.8 (15.6)	*t* = 2.268*
MATRICS composite score	33.9 (13.2)	29.1 (9.6)	*t* = 1.621
MATRICS working memory	41.0 (10.6)	35.0 (8.1)	*t* = 2.527*
MATRICS processing speed	38.6 (13.0) \	36.0 (10.3)	*t* = 0.895
Clinical, *M*(*SD*)			
BPRS mean item score—all items	1.7 (0.4)	1.7 (0.5)	*t* = 0.334
BPRS mean item score—Psychosis	2.1 (1.2)	2.3 (1.2)	*t* = 0.663
BPRS mean item score—Depression	1.8 (0.9)	2.1 (1.1)	*t* = 1.180
SANS mean item score—all items	1.5 (0.7)	1.5 (0.6)	*t* = 0.184
SANS mean item score—Avolition/Anhedonia	2.0 (0.9)	1.9 (0.7)	*t* = 0.557

*Note*. AA = ambiguity averse. AFA = African American. AS = Asian. BPRS = Brief Psychiatric Rating Scale. C = Caucasian. MATRICS = Measurement and Treatment Research to Improve Cognition in Schizophrenia Consensus Cognitive Battery. M/O = mixed/other. SANS = Scale for the Assessment of Negative Symptoms. WASI = Wechsler Abbreviated Scale of Intelligence. WRAT-Reading = Wide-Ranging Achievement Test, Reading Subtest. WTAR = Wechsler Test of Adult Reading.

a *n* = 85.

b *n* = 23.

* *p* < 0.05.

** *p* < 0.01.

*** *p* < 0.001.

**Table 3.** Analyses of correlations between model-free and model-based measures of experimental behavior and clinical variables in patients in the Horizon Task

Variable	BPRS overall mean	BPRS Psychosis item mean	BPRS Depressio item mean	SANS overall mean	SANS Avolition/Anhedonia item mean
Model-based measures of performance					
Prior mean	0.03	0.02	−0.09	0.05	0.10
Initial learning rate	−0.25**	−0.28**	−0.10	−0.06	−0.08
Asymptotic learning rate	0.08	0.09	0.03	−0.03	−0.03
Model-free measures of information seeking/directed exploration					
Overall information seeking [Σ*p*(high info)]	0.01	0.01	−0.04	0.02	0.06
Directed exploration [Δ*p*(high info)]	0.06	0.03	0.12	−0.05	−0.06
Model-based measures of information seeking/directed exploration					
Information weight (Horizon 1)	−0.02	−0.01	−0.14	0.04	0.08
Information weight (Horizon 6)	0.01	0.05	0.01	−0.11	−0.06
Change in information weight	0.02	0.08	0.16	−0.19*	−0.15
Model-free measures of behavioral variability/random exploration					
Overall behavioral variability [Σ*p*(low mean)]	0.17	0.23*	0.02	−0.01	−0.02
Random exploration [Δ*p*(low mean)]	0.29**	0.39***	0.19	−0.03	−0.03
Model-based measures of behavioral variability/random exploration					
Reward weight (Horizon 1 [1 3])	−0.03	−0.10	−0.04	−0.01	0.04
Reward weight (Horizon 6 [1 3])	−0.04	−0.17	−0.09	0.13	0.14
Reward weight (Horizon 1 [2 2])	0.09	0.11	0.08	0.11	0.12
Reward weight (Horizon 6 [2 2])	−0.25*	−0.29**	−0.20*	0.11	0.11
Change in reward weight ([1 3])	0.01	−0.01	−0.09	0.14	0.12
Change in reward weight ([2 2])	−0.15	−0.20*	−0.15	−0.02	−0.02

*Note*. Correlation values are Spearman coefficients. Correlations illustrated in Figure 6 are **bolded**. BPRS = Brief Psychiatric Rating Scale. SANS = Scale for the Assessment of Negative Symptoms.

* *p* < 0.05.

** *p* < 0.014.

*** *p* < 0.001.

**Table 4.** Correlations between measures of experimental behavior and cognitive variables in patients in the Horizon Task

Construct	Overall Performance	Overall Information Seeking	Directed Exploration	Overall Behavioral Variability	Random Exploration
Variable	Prior mean	Σ*p*(high info)	Δ*p*(high info)	Σ*p*(low mean)	∆*p*(low mean)
WASI estimated IQ (four subtests)	0.385***	0.387***	0.227*	−0.292**	0.029
WTAR scaled score	0.248**	0.223*	0.210	−0.317***	0.102
MATRICS composite score	0.303**	0.351***	0.212*	−0.300**	0.001
MATRICS domain scores					
Working Memory	0.292**	0.355***	0.186	−0.211*	0.079
Processing Speed	0.259**	0.276**	0.128	−0.239*	−0.024
Attention/Vigilance	0.194*	0.257**	0.156	−0.295**	−0.070
Verbal Learning	0.257**	0.336***	0.232*	−0.059	0.189*

*Note*. Correlation scores are Spearman correlation coefficients; correlations illustrated in Figure 6 are **bolded**. MATRICS = Measurement and Treatment Research to Improve Cognition in Schizophrenia Consensus Cognitive Battery. WASI = Wechsler Abbreviated Scale of Intelligence. WTAR = Wechsler Test of Adult Reading

* *p* < 0.05.

** *p* < 0.01.

*** *p* < 0.001.
