# Supplementary material for: Differential Effects of Psychotic Illness on Directed and Random Exploration
Source: Comput Psychiatr. Author manuscript; Available in PMC 2021 Mar 24. (PMC7990386; doi:10.1162/cpsy_a_00027)
Supplement: Supplementary information [file NIHMS1644471-supplement-Supplementary_information.pdf]

Supplemental Materials for:

Differential effects of psychotic illness on directed and random exploration

Waltz, J. A.[1*+], Wilson, R. C.[2, 3*], Albrecht, M. A.[1,4], Frank, M. J.[5,6], Gold, J. M.[1]

1. Maryland Psychiatric Research Center, University of Maryland School of Medicine, Baltimore, MD, USA
2. Department of Psychology, University of Arizona, Tucson, AZ, USA
3. Cognitive Science Program, University of Arizona, Tucson, AZ, USA
4. School of Public Health, Curtin Health Innovation Research Institute, Curtin University, Perth, Western Australia, Australia
5. Department of Cognitive, Linguistic and Psychological Sciences, Brown University, Providence, Rhode Island, USA
6. Department of Psychiatry and Brown Institute for Brain Science, Brown University, Providence, Rhode Island, USA

* These authors contributed equally
[+] To whom correspondence should be addressed:

James A. Waltz, PhD
University of Maryland School of Medicine
Maryland Psychiatric Research Center
P.O. Box 21247
Baltimore, MD 21228
E-mail: jwaltz@som.umaryland.edu

Supplemental Text: 2,569 words

Supplemental Figures (6)

1. Summary of k-means results for p(high info).
2. Summary of Gaussian Mixture Model results for p(high info).
3. Results of the model-based analysis: AA patients vs. non-AA (main) patients
4. Horizon Task performance in non-ambiguity-averse subjects (patients vs. controls).
5. Results of the model-based analysis: non-AA (main) group patients vs. non-AA (main) group controls.
6. Parameter recovery for the hierarchical Bayesian model

Supplemental Tables (5)

1. Demographic and cognitive variables in non-AA patients and non-AA controls.
2. Parameters of Hierarchical Bayesian Model.
3. Analyses of correlations between cognitive variables and model-based measures of experimental behavior from the Horizon Task in patients
4. Analyses of correlations between cognitive variables and model-free measures of directed and random exploration from the Horizon Task in controls
5. Analyses of correlations between cognitive variables and model-based measures of experimental behavior from the Horizon Task in controls

Supplementary Methods

Participant Medications

The vast majority (100/108) of participants with schizophrenia or schizoaffective disorder (collectively called "SZ") were medicated with antipsychotic drugs (APDs). Of the 100 patients taking APDs, most (66) were taking a single second-generation APD; the remaining 34 medicated patients were taking either a single first-generation APD (8) or a combination of APDs (26). Of the 66 patients taking a single second-generation APD, the majority (35) were taking clozapine; of the remaining 31 patients were taking a single second-generation APD, 9 were taking risperidone/paliperidone, 7 were taking olanzapine, 5 were taking aripiprazole, 4 were taking quetiapine, 3 were taking lurasidone, 2 were taking ziprasidone, and 1 was taking cariprazine.

Clustering analysis to identify the ambiguity averse group

In the paper we defined the ambiguity averse group by eye using a cutoff of p(high info) < 0.25 for both horizons. This defined a region of behavioral space we had never observed before using this task, and separated out what, by eye, appears to be two clusters of participants – ambiguity averse and non-ambiguity averse. To test whether this by-eye clustering was supported by the data, we performed two clustering analyses (1) a k-means clustering approach and (2) a Gaussian mixture model. The upshot of these analysis is that our by-eye heuristic aligns very well with these more principled clustering methods, with our heuristic being more conservative than k-means (which puts 2 more subjects in the AA group) and less conservative than Gaussian mixtures (which puts 3 fewer subjects in the AA group). For this reason, we retain the original heuristic as the cutoff in the paper.

For the k-means analysis we used the kmeans function in Matlab with 100 different random starting conditions (to reduce the chance of finding a local minimum). To determine the "correct" number of clusters we used the elbow (Thorndike, 1953) and silhouette (Rousseeuw, 1987) methods. In the elbow method (Figure S1, top left) we compute the square distance between each data point and the center of the cluster it is assigned to. By definition, this distance decreases monotonically as the number

of clusters increases. The “correct” number of clusters is determined by eye as the point at which the decrease in distance with k flattens out (i.e. at the “elbow” of the plot). For our data, this occurs at k = 2.

In the silhouette method, we compute a silhouette score for each value of k greater than or equal to 2. To compute this score, we first compute a silhouette score for each data point based on the mean distance between this data point and all other points in its cluster, a, and the mean distance between this data point and all other points in the next-nearest cluster, b. The silhouette score for this data point is then given as

$$S = \frac{b - a}{\max(a, b)}$$

The silhouette score for the clustering is simply the average silhouette score over all data points. This score varies from +1 for good clustering, to -1 for poor clustering, and in most cases will peak (as a function of k) at the “best” clustering level. In our case (Figure S1, top right) the highest silhouette score occurs at k = 2. Note, that the silhouette score is undefined for k = 1 (because there is no next-nearest cluster making b in the above equation undefined) so the silhouette method rules out k > 2 but does not rule out k = 1. However, combined with the elbow method we conclude that the optimal value of k for k-means clustering is k = 2.

What does the k = 2 clustering look like? As shown in the Figure S1 (bottom left) it closely resembles our original heuristic clustering (grey shaded area). Likewise, the k = 3 clustering also pulls out a similar ambiguity averse group as one cluster (Figure S1, bottom right).

As an additional check of the clustering, we also used a Mixture-of-Gaussians approach. These models can be thought of as a generalization of k-means that allow for the possibility of anisotropic clusters. In particular, these models assume that the data are generated as samples from a weighted sum of k Gaussians (in this case two-dimensional Gaussians) with arbitrary covariance matrices. For a given number, k, of Gaussians, the model estimates the mean and covariance of the Gaussians as well as their mixing weights. The “correct” number of clusters can be assessed using the Bayes Information Criterion (BIC; Schwarz, 1978) as well as the silhouette method.

Applied to the p(high info) data, we find that the BIC score is greatly improved going from 1 to 2 clusters and slightly improved by including a third cluster (Figure S2, top left). Beyond k = 3, BIC increases again due to overfitting. While BIC suggests that k = 3 is the best solution, the silhouette method (Figure S2, top right) again suggests k = 2 is the best solution. Crucially, both the k = 2 and k = 3 solutions identify the AA group as a separate cluster (with one subject differing in assignment between the k = 2 and k = 3 solutions). Again, the clustering is similar to our original heuristic (grey area, Figure S2, bottom). For simplicity we therefore focus on the k = 2 solution.

Details of Modeling Analysis

To further quantify the differences in behavior between the different groups (controls vs patients) and subgroups (non-AA patients vs AA patients), we turned to computational modeling. In particular we fit the model of Zajkowski et al. (2017) to the behavioral data of three groups (non-AA group controls, non-AA group patients, and ambiguity-averse patients (excluding the two ambiguity averse controls). In addition to quantifying directed and random exploration, via the information weight, β_I, and reward weight, β_R, respectively, this model also allows us to quantify the learning process with three parameters: a prior mean, R_0, and two learning rates corresponding to the initial learning rate,α_1, and asymptotic learning rate, α_∞. The model (illustrated in Figure 2 of the main text) naturally decomposes into a learning component and a decision component. We consider each of these components in turn, in the main text.

Model Fitting

Hierarchical Bayesian Model

Between the learning and decision components of the model, each subject's behavior is described by 13 free parameters. These parameters are: the initial mean, R_0, the initial learning rate, α_1, the asymptotic learning rate, α_∞, the information bonus, A, in both horizon conditions, the spatial bias, B, in

the four horizon x uncertainty conditions, and the decision noise, σ, in the four horizon x uncertainty conditions.

Each of the free parameters is fit to the behavior of each subject using a hierarchical Bayesian approach (Lee & Wagenmakers, 2014). In this approach to model fitting, each parameter for each subject is assumed to be sampled from a group-level prior distribution whose parameters, the so-called "hyperparameters", are estimated using a Markov Chain Monte Carlo (MCMC) sampling procedure. The hyper-parameters themselves are assumed to be sampled from "hyperprior" distributions whose parameters are defined such that these hyperpriors are broad. For notational convenience, we refer to the hyperparameters that define the prior for variable X as θ^X. In addition, we use subscripts to refer to the dependence of both parameters and hyperparameters on the horizon condition,h, uncertainty condition, u, subject, s, and game, g. In addition, in order to understand the differences between the three different groups of subjects (non-AA group; see below) controls and patients and ambiguity averse patients), we also allow the parameters and hyperparameters to vary by group, G.

The particular priors and hyperpriors for each parameter are shown in Table S2. For example, we assume that the prior mean, R_0^{Gs}, for each subject group G and horizon condition h, is sampled from a Gaussian prior with mean $\mu_{R_0}^G$ and standard deviation $\sigma_{R_0}^G$. These prior parameters are sampled in turn from their respective hyperpriors: $\mu_{R_0}^G$, from a Gaussian distribution with mean 50 and standard deviation 14, $\sigma_{R_0}^G$ from a Gamma distribution with shape parameter 1 and rate parameter 0.001.

Parameter transformation to facilitate presentation of results

The above model and fitting procedure are almost identical to that used in (Zajkowski et al., 2017), with the only difference being that we consider different groups of subjects rather than different transcranial magnetic stimulation conditions within subject. While this fitting procedure performs well on the schizophrenia data set, the presence of a new behavioral phenotype (extreme ambiguity aversion – see below) leads to parameter values that are difficult to visualize (e.g. an information bonus of -200 points,

which is an order of magnitude larger than anything we have previously observed). For this reason, while the model is fit using the parameterization described above, we have found it useful to present the results in a transformed parameter set. This transformed model writes the decision rule as

$$p(choose\ right) = \frac{1}{1 + exp(\beta_R \Delta R + \beta_I \Delta I + \beta_S)}$$

where the reward weight, β_R, the information weight, β_I, and spatial bias weight β_S, are related to σ, A, and B as follows:

$$\beta_R = \frac{1}{\sigma}$$

$$\beta_I = \frac{A}{\sigma}$$

$$\beta_S = \frac{B}{\sigma}$$

Supplementary Results

Comparison of non-AA controls and non-AA patients on experimental measures

Among non-AA participants, there was a trend toward a main effect of diagnostic group on performance ($F_{1,570}$ = 3.67, p = 0.058), suggestive of reduced performance relative to community controls. The interaction between group and trial was not significant ($F_{5,570}$ = 0.57, p = 0.721), however.

Next, we asked whether non-AA controls and non-AA patients showed differential effects of horizon on directed exploration, finding that the interaction between group and horizon trended toward significance ($F_{1,114}$ = 2.81, p = 0.059). A post-hoc t-test directly comparing non-AA patients and non-AA controls revealed a significant between-group difference in the proportion of high-information choices at horizon 6 (two-sided t_{114} = 2.69, p = 0.008). For random exploration, we observed a trend toward a significant main effect of diagnostic group (non-AA controls vs. non-AA patients) on overall variability ($F_{1,114}$ = 3.75, p = 0.06), but no interaction between subject type and horizon ($F_{1,114}$ = 0.20, p = 0.66).

As expected, non-AA patients and non-AA controls differed in their levels of educational attainment and on multiple measures of cognitive performance (Table S2).

Model-based Analyses of directed and random exploration

The left-hand column of Figure S3 shows individual parameter values for patients in the AA and non-AA (main) subgroups. In the right-hand column, the distributions over the difference in parameters between the two patient groups are plotted. Consistent with model-free measures of directed and random exploration, model-based analyses indicated that AA patients showed no evidence for information seeking in any context. Specifically, for the ambiguity averse patients, we observed reduced information weighting in both horizon 1 and horizon 6, relative to non-AA group patients (100% of samples less than zero, respectively). In addition, ambiguity averse patients showed much reduced reward weight in the [1 3] condition (100% of samples), which is almost zero for most people in this group. Both results are consistent with the extreme ambiguity aversion in this group and suggest that AA patients base their decision almost exclusively on avoiding the uncertain option in the [1 3] condition. Interestingly, reward weight does not appear to differ between non-AA and AA patients in the [2 2] condition, consistent with the ability of AA patients to perform quite well in this condition.

The left-hand column of Figure S5 shows individual parameter values for patients in the non-AA (main) subgroup and controls in the non-AA (main) subgroup. In the right-hand column, the distributions over the difference in parameters between the two groups are plotted. Also consistent with the model-free measures, non-AA patients exhibited reduced directed exploration relative to non-AA controls, as indicated by a reduction in the information weight, β_I, in horizon 6 (96.5% of samples for the mean information weight of non-AA group patients below those for non-AA group controls).

Parameter recovery analysis

To test whether the fitting procedure and experiment were sufficient to estimate reliable parameter values, we performed a parameter recovery analysis (Wilson & Collins, 2019). In this analysis, the fitting procedure is performed on simulated data, allowing us to compare the "true" parameters used

in the simulations with the fit parameters found by model fitting. If the fitting procedure is robust, and the experiment sufficiently powered, then the fit parameters should closely match the true parameters.

In particular, we generated "fake" experimental data by simulating choices from 141 people using the parameter values obtained by fitting real subjects. We then fit this fake data with the same model that we used to analyze the real data and then tested whether the true and fit parameter values were related using a simple correlation. As shown in Figure S6, parameter identifiability is high, with correlations between simulated and recovered parameters over 0.8 for all parameters except the asymptotic learning rate, where recoverability is hampered by the narrow range of fit parameter values across people. This shows that parameters from simulated data are recoverable.

Analyses of correlations between model-based parameters quantifying directed and random exploration and cognitive variables in patient participants

We observed significant correlations between the information weight in horizon 6 and most cognitive measures. In addition, we observed significant correlations between the change in information weight between horizon 1 and horizon 6 and most cognitive measures. Where the model-based measures differ from the model-free measures is in the information weight in horizon 1, where we see no significant correlations with information weight. At first sight, this result appears at odds with results from the model-free analyses, where the cognitive variables were found to correlate with p(high info) in both horizon conditions. However, in the model, choosing the more informative (and also more uncertain) option is driven not only by the information weight, but also by the prior mean, R_0 (with lower R_0 associated with increased ambiguity aversion) and initial learning rate, α_1 (with smaller α_1 associated with a stronger effect of the prior mean). In this regard, it is notable that both of these parameters exhibit numerically positive correlations with several cognitive variables. This suggests that the correlation between cognitive variables and p(high info) in horizon 1 may be driven by correlations between these variables and the prior.

For behavioral variability, we found significant negative correlations between most cognitive variables and the model-free measures of behavioral variability, p(low mean), in both horizon conditions. Thus, patients who were more random overall, scored lower on most cognitive tests. Unlike information seeking, however, these correlations were of similar size in both horizon 1 and horizon 6, and there were no significant correlations between the change in p(low mean) with horizon and any cognitive variable, suggesting no association with random exploration. These model-free findings were consistent with the model-based results. In particular we find positive correlations between the reward weight in both horizon conditions and most cognitive variables (note that increased reward rate is associated with *less* behavioral variability), but only one significant correlation with the change in reward weight with horizon (between MATRICS Verbal Learning scores and the change in reward weight in the [2 2] condition).

Model-free measures of behavior on the later trials in the horizon 6 games was also correlated with cognitive variables. Information seeking scores [p(high info)] for all trials in the horizon 6 games were significantly correlated with MATRICS Verbal Learning scores, suggesting a strong association between the likelihood of engaging in directed exploration and verbal learning abilities. For behavioral variability, almost all cognitive variables were negatively correlated with random exploration scores [p(low mean)] across trials.

Table S1. Demographic and cognitive variables in non-AA patients and non-AA controls.

Measure	Non-AA Patients (N=85) Mean (SD)	Non-AA Controls (N=31) Mean (SD)	Inferential Statistic
**Demographic**			
Age	36.7 (10.3)	35.8 (10.5)	t=0.400
Gender	26 F, 59 M	10 F, 21 M	χ^2=0.030
Race	47 C, 27 AA, 3 AS, 7 M/O	17 C, 12 AA, 0 AS, 2 M/O	χ^2=1.503
Subject Education	13.3 (2.1)	15.2 (2.0)	t=4.365***
Parental Education	14.6 (2.7)	14.1 (2.4)	t=0.797
**Cognitive**			
WASI Estimated IQ (4 subtests)	96.1 (14.7)	112.2 (13.8)	t=5.301***
WRAT-Reading Scaled Score	98.6 (15.5)	109.3 (14.9)	t=3.318**
WTAR Scaled Score	101.0 (17.6)	110.5 (14.3)	t=2.997**
MATRICS Composite Score	33.9 (13.2)	51.9 (11.1)	t=6.784***
MATRICS Working Memory	41.0 (10.6)	52.4 (11.8)	t=4.960***
MATRICS Processing Speed	38.6 (13.0)	53.5 (12.0)	t=5.550***

Table S2. Parameters of Hierarchical Bayesian Model.

Parameter	Prior	Hyperparameters	Hyperpriors
prior mean, R_0^{Gs}	$R_0^{Gs} \sim Gaussian(\mu_{R_0}^G, \sigma_{R_0}^G)$	$\theta_{R_0}^G = (\mu_{R_0}^G, \sigma_{R_0}^G)$	$\mu_{R_0}^G \sim Gaussian(50,14)$<br>$\sigma_{R_0}^G \sim Gamma(1,0.001)$
initial learning rate, α_1^{Gs}	$\alpha_1^{Gs} \sim Beta(a_{\alpha_1}^G, b_{\alpha_1}^G)$	$\theta_{\alpha_1}^G = (a_{\alpha_1}^G, b_{\alpha_1}^G)$	$a_{\alpha_1}^G \sim Uniform(0.1,10)$<br>$b_{\alpha_1}^G \sim Uniform(0.5,10)$
asymptotic learning rate, α_∞^{Gs}	$\alpha_\infty^{Gs} \sim Beta(a_{\alpha_1}^G, b_{\alpha_1}^G)$	$\theta_{\alpha_\infty}^G = (a_{\alpha_\infty}^G, b_{\alpha_\infty}^G)$	$a_{\alpha_\infty}^G \sim Uniform(0.1,10)$<br>$b_{\alpha_\infty}^G \sim Uniform(0.1,10)$
information bonus, A^{Gshu}	$A^{Gshu} \sim Gaussian(\mu_A^{Ghu}, \sigma_A^{Ghu})$	$\theta_A^{Ghu} = (\mu_A^{Ghu}, \sigma_A^{Ghu})$	$\mu_A^{Ghu} \sim Gaussian(0,100)$<br>$\sigma_A^{Ghu} \sim Gamma(1,0.001)$
spatial bias, B^{Gshu}	$B^{Gshu} \sim Gaussian(\mu_B^{Ghu}, \sigma_B^{Ghu})$	$\theta_B^{Ghu} = (\mu_B^{Ghu}, \sigma_B^{Ghu})$	$\mu_B^{Ghu} \sim Gaussian(0,100)$<br>$\sigma_B^{Ghu} \sim Gamma(1,0.001)$
decision noise, σ^{Gshu}	$\sigma^{Gshu} \sim Gamma(k_\sigma^{Ghu}, \lambda_\sigma^{Ghu})$	$\theta_\sigma^{Ghu} = (k_\sigma^{Ghu}, \lambda_\sigma^{Ghu})$	$k_\sigma^{Ghu} \sim Exp(0.1)$<br>$\lambda_B^{Ghu} \sim Exp(10)$

Table S3. Correlations between cognitive variables and model-free measures of directed and random exploration from the Horizon Task in controls.

Construct	Overall Information Seeking	Directed Exploration	Overall Behavioral Variability	Random Exploration
**Variable Name**	**Σp(high info)**	**Δp(high info)**	**Σp(low mean)**	**Δp(low mean)**
**Estimated IQ (from 4-subtest WASI)**	0.371*	0.093	-0.151	-0.098
**WTAR Scaled Score**	0.256	0.165	-0.328	0.012
**MATRICS Composite Score**	0.369*	0.120	-0.347*	-0.237
**MATRICS Domain Scores**				
**- Working Memory**	0.276	0.053	-0.269	-0.166
**- Processing Speed**	0.318	0.143	-0.324	-0.300
**- Attention & Vigilance**	0.295	0.139	-0.371*	-0.088
**- Verbal Learning**	0.301	0.478**	0.139	0.220

Correlation scores are Spearman correlation coefficients; * indicates $p < 0.05$, ** $p < 0.01$.

Table S3. Analyses of correlations between model-based measures of experimental behavior from the Horizon Task and cognitive variables in patients.

Variable Name	WASI Estimated IQ	WTAR Scaled Scare	MATRICS Composite Score	MATRICS WM Subscore	MATRICS Processing Speed Subscore
**Model-based measures of Performance**					
**prior mean**	0.385***	0.248**	0.303**	0.292**	0.259**
**initial learning rate**	0.180	0.180	0.132	0.093	0.094
**asymptotic learning rate**	0.295**	0.148	0.235*	0.135	0.238*
**Model-based measures of information seeking/ directed exploration**					
**information weight (horizon 1)**	0.056	-0.083	-0.027	0.031	-0.002
**information weight (horizon 6)**	0.174	0.130	0.169	0.189	0.106
**change in information weight**	0.063	0.165	0.116	0.068	0.064
**Model-based measures of behavioral variability/ random exploration**					
**reward weight (horizon 1 [1 3])**	-0.372***	-0.441***	-0.338***	-0.329***	-0.257**
**reward weight (horizon 6 [1 3])**	-0.372***	-0.317***	-0.253**	-0.260**	-0.200*
**reward weight (horizon 1 [2 2])**	-0.332***	-0.384***	-0.286**	-0.325***	-0.204*
**reward weight (horizon 6 [2 2])**	-0.183	-0.146	-0.220*	-0.204*	-0.190*
**change in reward weight ([1 3])**	-0.251**	-0.132	-0.096	-0.150	-0.082
**change in reward weight ([2 2])**	-0.024	0.036	-0.055	-0.050	-0.050

Correlation values are Spearman coefficients; * indicates $p < 0.05$, ** $p < 0.01$, *** $p < 0.001$.

Table S5. Analyses of correlations between model-based measures of experimental behavior from the Horizon Task and cognitive variables in controls.

Variable Name	WASI Estimated IQ	WTAR Scaled Scare	MATRICS Composite Score	MATRICS WM Subscore	MATRICS Processing Speed Subscore
**Model-based measures of Performance**					
**prior mean**	0.396*	0.357*	0.324	0.304	0.325
**initial learning rate**	0.155	0.350*	0.257	0.296	0.233
**asymptotic learning rate**	0.093	0.177	0.081	0.127	0.069
**Model-based measures of information seeking/ directed exploration**					
**information weight (horizon 1)**	0.144	-0.205	0.070	0.040	0.037
**information weight (horizon 6)**	0.216	0.025	0.210	0.147	0.178
**change in information weight**	0.164	0.201	0.216	0.159	0.201
**Model-based measures of behavioral variability/ random exploration**					
**reward weight (horizon 1 [1 3])**	-0.238	-0.247	-0.315	-0.256	-0.248
**reward weight (horizon 6 [1 3])**	-0.253	-0.025	-0.250	-0.202	-0.173
**reward weight (horizon 1 [2 2])**	-0.042	-0.204	-0.185	-0.113	-0.164
**reward weight (horizon 6 [2 2])**	-0.130	-0.046	-0.295	-0.288	-0.256
**change in reward weight ([1 3])**	-0.093	0.188	-0.022	-0.016	0.012
**change in reward weight ([2 2])**	-0.120	0.016	-0.243	-0.259	-0.211

Correlation values are Spearman coefficients; * indicates $p < 0.05$.

Figure S1 – Summary of k-means results for p(high info). Top: both elbow and silhouette methods suggest that the k = 2 solution is best. Bottom: best fitting clustering solutions for k = 2 and k = 3. Shaded grey area corresponds to criteria for AA group used in original submission (i.e. p(high info) < 0.25 for both horizon conditions).

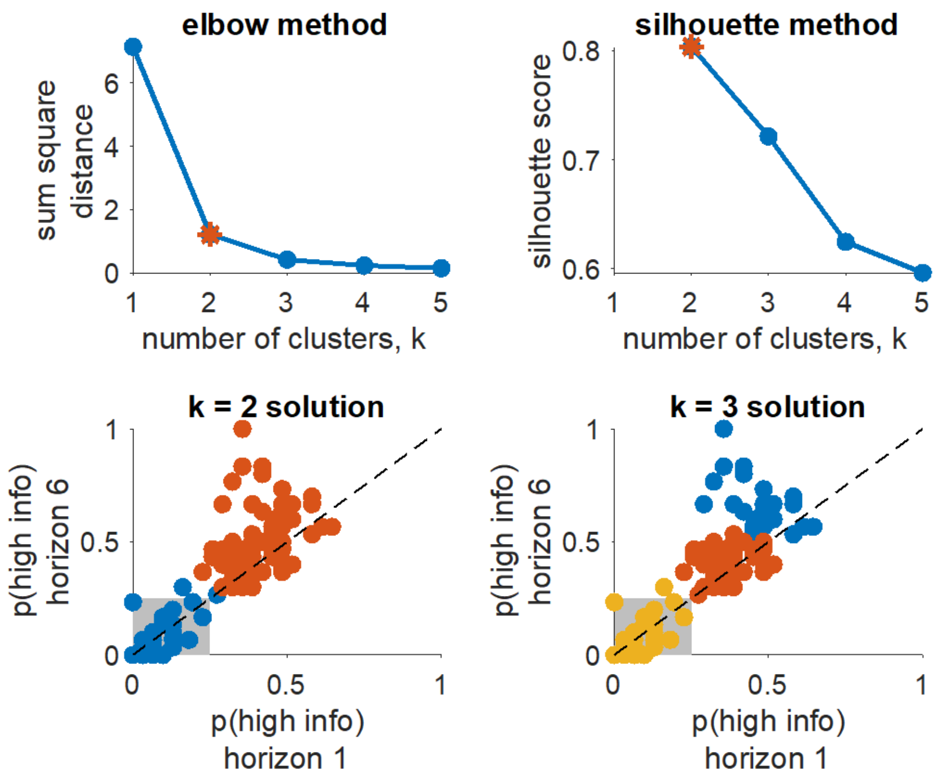

Figure S2 – Summary of Gaussian Mixture Model results for p(high info). Top: The BIC method suggests that k = 3 is the best solution, while the silhouette methods again suggest that the k = 2 solution is best. Bottom: best fitting clustering solutions for k = 2 and k = 3. Shaded grey area corresponds to criteria for AA group used in original submission (i.e. p(high info) < 0.25 for both horizon conditions).

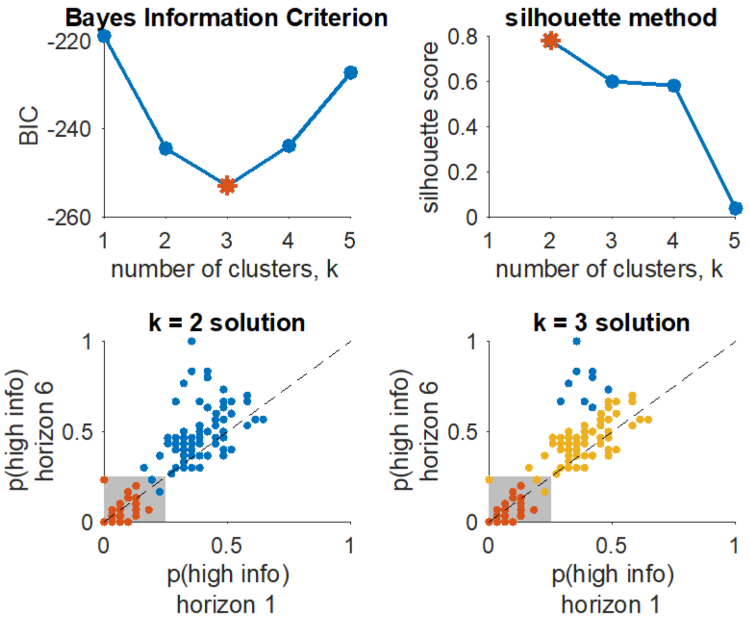

Figure S3 – Results of the model-based analysis: AA patients vs. non-AA (main) patients. (A) Subject-level parameter values. (B) Posterior distributions over the difference in group-level parameters between AA patients and non-AA-group patients.

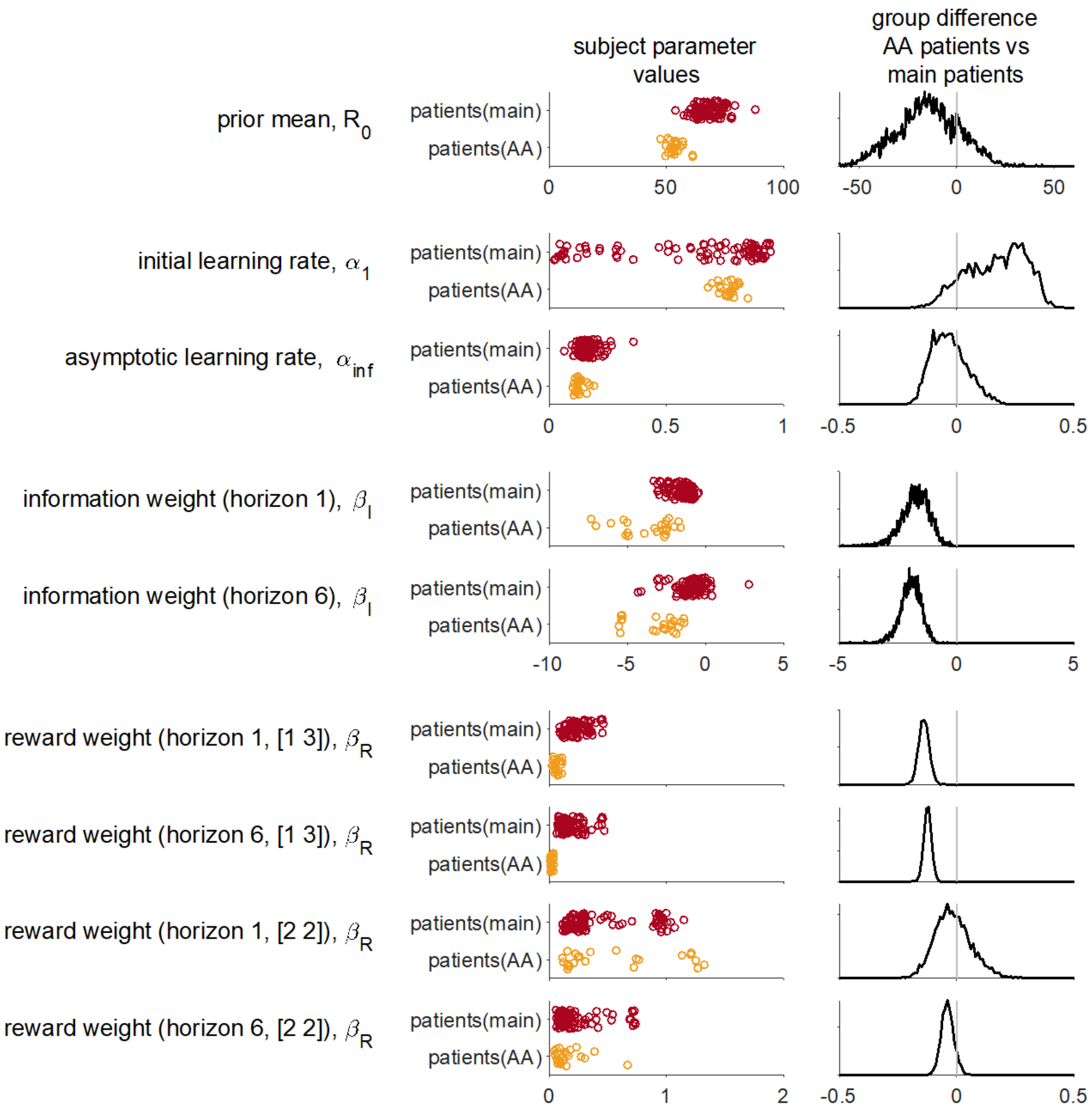

Figure S4 – Horizon Task performance in non-ambiguity-averse subjects (patients vs. controls). (A) Overall Horizon Task performance in the Non-AA (main) group of patients and the Non-AA (main) group of controls. (B) Directed Exploration in Non-AA patients and Non-AA controls. (C) Random Exploration in Non-AA patients and Non-AA controls.

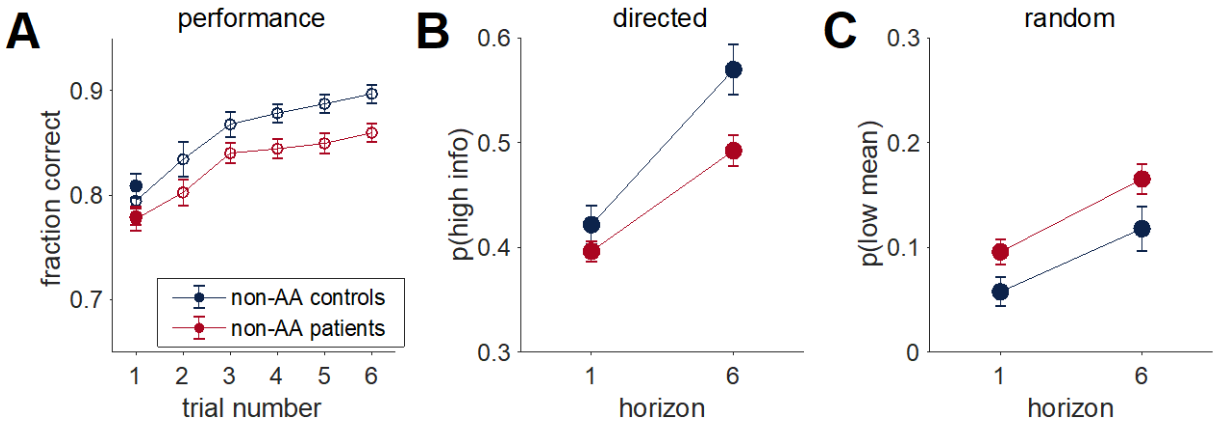

Figure S5 – Results of the model-based analysis: non-AA (main) group patients vs. non-AA (main) group controls. (A) Subject-level parameter values. (B) Posterior distributions over the difference in group-level parameters between non-AA patients and controls.

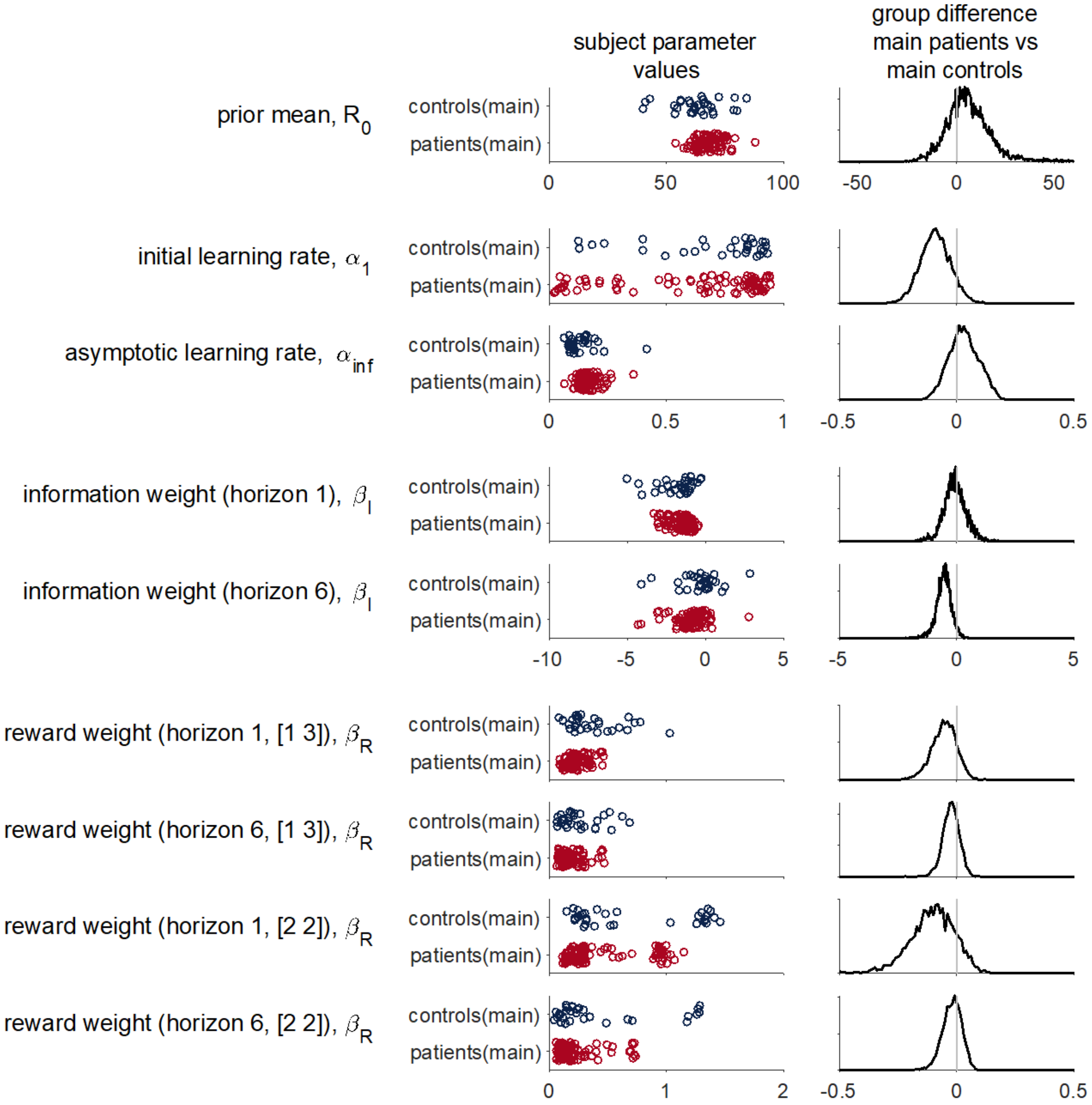

Figure S6 – Parameter recovery for the hierarchical Bayesian model. Each panel corresponds to a different model parameter and compares the simulated value of that parameter (i.e. the ground truth) to the fit parameter value. The parameter values for the simulated data were identical to those found by fitting the real data, thus there were exactly the same number of participants in the simulated data set as the real data set (139 split into three groups, non-AA controls, non-AA patients, and AA patients; two AA controls were not included). In addition, the simulated data set was based on the exact task (i.e. pattern of forced choices, observed rewards in forced choices, horizon conditions, etc.) seen by the real participants. Good parameter recovery is indicated by a strong correlation between simulated and fit parameter values.

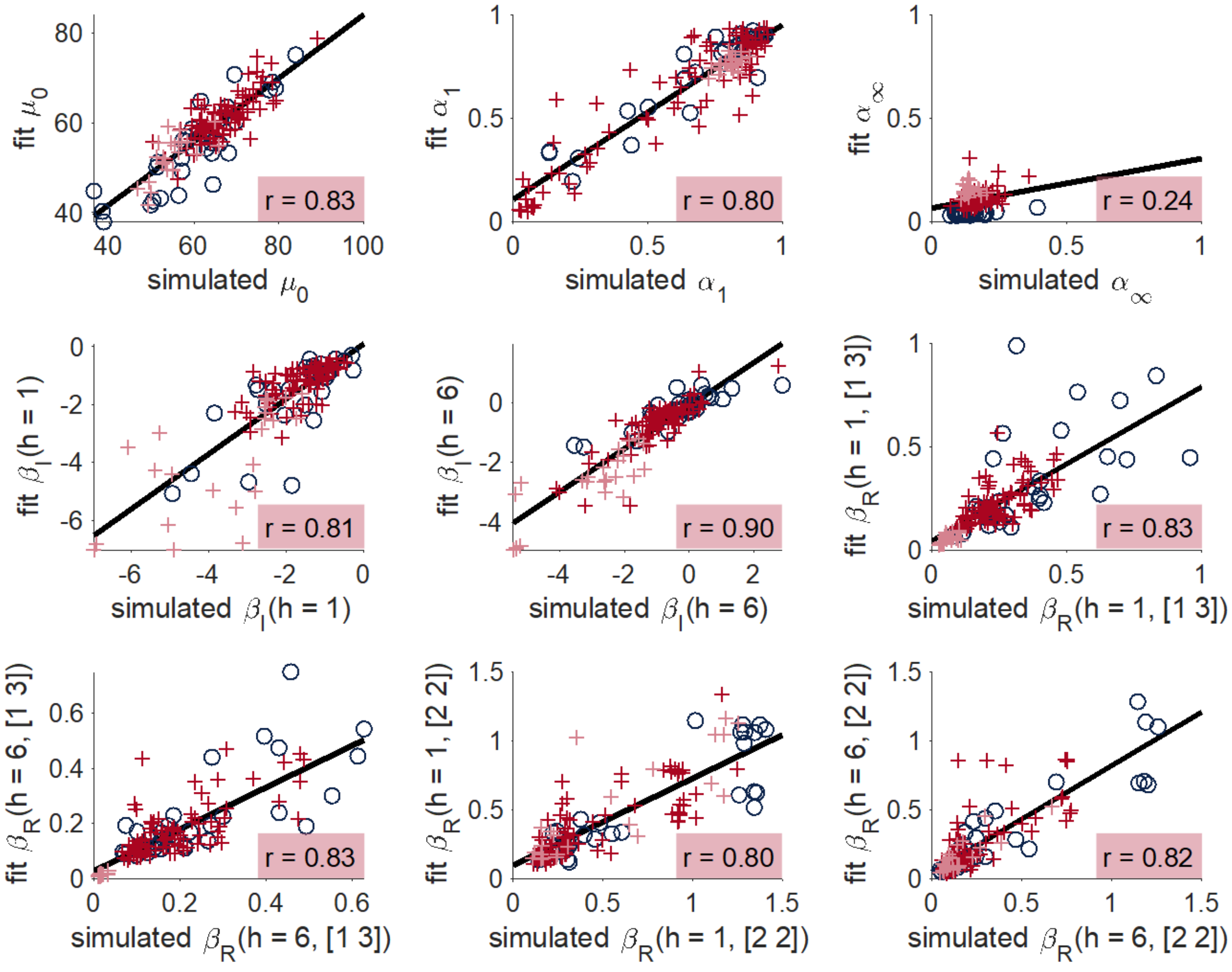

References

Lee, M. D., & Wagenmakers, E.-J. (2014). *Bayesian cognitive modeling: A practical course*: Cambridge university press.

Rousseeuw, P. J. (1987). Silhouettes: a graphical aid to the interpretation and validation of cluster analysis. *Journal of computational and applied mathematics, 20*, 53-65.

Schwarz, G. (1978). Estimating the dimension of a model. *The annals of statistics, 6*(2), 461-464.

Thorndike, R. L. (1953). Who belongs in the family.

Wilson, R. C., & Collins, A. G. E. (2019). Ten simple rules for the computational modeling of behavioral data. *Elife, 8*, e49547.

Zajkowski, W. K., Kossut, M., & Wilson, R. C. (2017). A causal role for right frontopolar cortex in directed, but not random, exploration. *Elife, 6*.
